# Role of nuclear protein Akirin in the modulation of female reproduction in *Nilaparvata lugens* (Hemiptera: Delphacidae)

**DOI:** 10.3389/fphys.2024.1415746

**Published:** 2024-07-09

**Authors:** Feiyan Gou, Daowei Zhang, Siqi Chen, Mingjing Zhang, Jing Chen

**Affiliations:** ^1^ College of Basic Medical Science, Zunyi Medical University, Zunyi, China; ^2^ School of Biological and Agricultural Science and Technology, Zunyi Normal University, Zunyi, China

**Keywords:** *Nilaparvata lugens*, Akirin, reproduction, vitellogenin, hormone

## Abstract

**Introduction:** Akirin as a highly conserved transcription factor, exerts a profound influence on the growth, development, immune response, and reproductive processes in animals. The brown planthopper (BPH), *Nilaparvata lugens*, a major pest in rice production in Asia, possesses high reproductive capacity, a critical factor contributing to reduced rice yields. The aims of this study were to demonstrate the regulatory role of Akirin in the reproduction of BPH.

**Methods:** In this study, quantitative PCR (qPCR) was used to detect the mRNA expression of genes. RNA interference (RNAi) was used to downregulate the expression of *Akirin* gene, and RNA sequencing (RNA-seq) was used to screen for differentially expressed genes caused by *Akirin* downregulation. Hormone contents were measured with the enzyme linked immunosorbent assay (ELISA), and protein content was evaluated with the bicinchoninic acid (BCA) method.

**Results:** Using BPH genome data, we screened for an *Akirin* gene (*NlAkirin*). An analysis of tissue-specific expressions showed that *NlAkirin* was expressed in all tissues tested in female BPH, but its expression level was highest in the ovary. After inhibiting the mRNA expression of *NlAkirin* in BPH females, the number of eggs laid, hatching rate, and number of ovarioles decreased. Transcriptome sequencing was performed, following a *NlAkirin* double-stranded RNA treatment. Compared with the genes of the control, which was injected with *GFP* double-stranded RNA, there were 438 upregulated genes and 1012 downregulated genes; the expression of *vitellogenin* (*Vg*) and *vitellogenin receptor* (*VgR*) genes as well as the mRNA expression of genes related to the target of rapamycin (TOR), juvenile hormone (JH), and insulin pathways involved in Vg synthesis was significantly downregulated. As a result of *NlAkirin* knockdown, the titers of JH III and Ecdysone (Ecd) were downregulated in unmated females but returned to normal levels in mated females. The ovarian protein contents in both unmated and mated females were downregulated.

**Discussion and conclusion:** Our results suggest that *NlAkirin* affects female BPH reproduction by regulating the mRNA expression of genes related to the *Vg*, *VgR*, TOR, JH, and insulin signaling pathways, in addition to the titers of JH III and Ecd. The findings of this research provide novel insights into the regulatory role of *Akirin* in insect reproductive capacity.

## 1 Introduction

*Akirin* was discovered by Goto et al. (2008) using whole-genome RNA interference (RNAi) screening in *Drosophila*. The Akirin protein is strictly localized in the nucleus, where it functions as a key nuclear protein and regulates innate immune responses by controlling the expression of genes influencing the NF-κB-mediated immune deficiency (Imd) signaling pathway (Ferrandon et al., 2007; Goto et al., 2008; Bonnay et al., 2014). Akirin is present only in animals (Carreón et al., 2012; Manzano-Román et al., 2012; Carpio et al., 2013); two Akirin proteins, Akirin1 and Akirin2, have been identified in vertebrates, whereas only one has been found in insects (Hou et al., 2013; Merino et al., 2013; Bosch et al., 2020). All Akirin proteins have nuclear localization signals (NLS) at the N-terminus of the protein sequence. Immunostaining with Akirin antibodies specific to endogenous markers has shown strong nuclear localization in fruit flies (Bonnay et al., 2014), nematodes (Polanowska et al., 2018), rats (Komiya et al., 2008), mice (Bosch et al., 2018), and humans (Leng et al., 2019), indicating that it is a highly conserved nuclear transcription factor.

In insects, Akirin affects the innate immune response, which is the primary line of defense against pathogenic bacteria (Medzhitov and Janeway, 1997; Shaw et al., 2017). Akirin and Akirin2 in invertebrates and vertebrates, respectively, both cooperate with NF-κB to induce downstream factor expression of the Imd and Tumor Necrosis Factor/Toll-like receptor signaling pathways. For example, *Akirin* knockdown significantly affects the innate immune-related signaling pathway in *Drosophila melanogaster* and *Bombyx mori*, which increases their sensitivity to bacterial infection (Bonnay et al., 2014; Hu et al., 2021). In mice, Akirin2 interacts with BAF60a/b/c to respond to immune effectors in macrophages, thereby upregulating interleukin *IL-6* expression (Tartey et al., 2014). The impact of Akirin on the immune functions of *Litopenaeus vannamei* (Hou et al., 2013), *Procambarus clarkia* (Xiong et al., 2021), *Sogatella furcifera* (Chen et al., 2018), *Ixodes scapularis* (de la Fuente et al., 2013), *Caenorhabditis elegans* (Bowman et al., 2020), and other species has been observed, in addition to developmental abnormalities such as tissue damage, nymphal metamorphosis failure, reduced vector capacity, decreased survival rate, and reduced reproductive fitness (Almazán et al., 2005; de la Fuente et al., 2006). Inhibition of *Akirin* expression using gene knockdown or RNAi techniques affects embryonic development in mice, fruit flies, mosquitoes, ticks, and nematodes (Goto et al., 2008; Qu et al., 2014; Bosch et al., 2018; 2019); muscle development in mice and ducks (Macqueen et al., 2010; Mobley et al., 2014; Sun et al., 2016); neural development in *Xenopus laevis* (Liu et al., 2017); and stress responses, feeding, and growth in ticks (Busby et al., 2012; Rahman et al., 2018). Moreover, *Akirin* inhibition causes apoptosis in human malignant glioma cells (Krossa et al., 2015) and changes B cell cycle progression in mice (Tartey et al., 2015). After downregulating the expression of *Akirin* in *Anopheles arabiensis*, *Dermanyssus gallinae* and *Haemaphysalis longicornis*, their reproductive ability was significantly impaired (Lima-Barbero et al., 2019; Lee et al., 2020; Letinic et al., 2021). Furthermore, in our prior research, we observed that the mRNA expression levels of *Akirin* in the ovaries and testes of white-backed planthoppers, *S*. *furcifera*, were notably higher than in other tissues (Chen et al., 2018). Hence, we preliminarily hypothesize that Akirin is intricately linked to the reproductive capacity of planthoppers.

Vitellogenin (Vg) in insects accumulates in the oocytes in the form of yolk, serving as both a prerequisite for oocyte maturation into mature eggs and assurance for embryonic development postoviposition (Wu et al., 2020). The synthesis of insect Vg, maturation of oocytes, and uptake of Vg by mature oocytes play pivotal roles in the reproductive development of female insects, which are regulated by crucial hormones [juvenile hormone (JH) and ecdysone (Ecd) (Roy et al., 2018; Santos et al., 2019)] and multiple signaling pathways. As nutrient sensors, the amino acid/Target of Rapamycin signaling pathway (AA/TOR) and insulin pathway (or insulin-like peptide signaling pathway, ILP) participate in JH and Ecd biosynthesis, interacting synergistically with JH and Ecd to regulate insect yolk formation (Zhu et al., 2020; Dong et al., 2021; Pan et al., 2022).

The brown planthopper (BPH), *Nilaparvata lugens* (Stål), is one of the most destructive rice pests, and its high reproductive capacity has been the main driver of its outbreaks in Asia (Dong et al., 2021). In this study, we investigated the effect of *Akirin* on the ovarian development and reproduction of BPH females and revealed the reproductive signaling pathway mechanism using RNAi technology. The aims of this study were to demonstrate the regulatory role of Akirin in the reproduction of BPH, enrich and develop the molecular basis of insect reproduction regulation, and provide a resource for the development of new pest control strategies targeting reproduction regulation.

## 2 Materials and methods

### 2.1 Experimental insects

BPH individuals were continuously reared by our research group. More than 30 generations were propagated on rice seedlings (variety: TN1) in 80-mesh wooden cages (50 cm × 50 cm × 50 cm) in an artificial climate chamber (temperature, 27°C ± 1°C; relative humidity, 75%–80%; photoperiod, 16 L:8 D).

### 2.2 Sequence and phylogenetic tree analysis

The Akirin sequence of BPH was obtained by aligning the Akirin sequence of *S*. *furcifera* (AVW83290.1) with the genome sequence of BPH (GCF_000757685.1). The protein molecular weight (MW) and isoelectric point (pI) were predicted using the ExPaSy ProtParam tool (https://web.expasy.org/protparam/). SignalP-5.0 was used to predict the protein signal peptide (http://www.cbs.dtu.dk/services/Signalp/). Subsequently, structural domain analysis was performed using InterPro (https://www.ebi.ac.uk/interpro/search/sequence/). The open reading frame (ORF) of the gene was identified using EditSeq in DNAStar. ClustalX was used to align the amino acid sequences, and a neighbor-joining (NJ) tree was constructed using the MEGA 7.0 phylogenetic analysis program.

### 2.3 Extraction of total RNA and synthesis of the first strand of cDNA

Total RNA was extracted from BPHs using TRIzol reagent (Invitrogen, Carlsbad, CA, United States). The RNA concentration and A260/A280 ratio were determined using a NanoDrop 2000 spectrophotometer (Thermo, Waltham, United States). In RNase-free centrifuge tubes, the following components were added following the instructions of the PrimeScript™ RT Reagent Kit (RR037A, TakaRa, Beijing, China) to prepare the RT reaction solution. Briefly, 2 μL of 5 × PrimeScript Buffer, 0.5 μL of PrimeScript RT Enzyme Mix I, 0.5 μL of oligo dT primer (50 μM), and 200 ng of total RNA (all in RNase-free H_2_O) were added to the reaction, resulting in a total volume of 10 μL. Subsequently, the reaction was performed at 37°C for 15 min (reverse transcription), 85°C for 5 s (inactivation of reverse transcriptase) and terminated at 4°C. Extracted RNA and cDNA were stored at −80°C or immediately used.

### 2.4 qPCR analysis

Quantitative PCR (qPCR) was employed to assess the mRNA expression of genes in this study. Except for tissue distribution detection, qPCR detection of all other mRNA was performed using the whole body of BPH. Primers were designed using Primer Premier 5.0, and *RPS11* (GenBank ID: ACN79505.1) was used as the internal reference (Yuan et al., 2014). qPCR was performed using TB Green Premix Ex Taq II (RR820A, TakaRa, Beijing, China) with the LightCycler96 instrument (Bio-Rad, California, United States). The reaction solution comprised 12.5 μL of TB Green Premix Ex Taq II (Tli RNaseH Plus) (2×), 1 μL of PCR forward primer (10 μM), 1 μL of PCR reverse primer (10 μM), 2 μL of cDNA, and 8.5 μL of sterilized water, resulting in a total volume of 25 μL. The thermal cycling conditions were as specified by the manufacturer: predegeneration for 30 s at 95°C, 40 cycles of 95°C for 5 s, and 60°C for 30 s. A standard curve was generated to test the amplification efficiency, which should have been over 90%. Amplification and melting curves of qPCR were obtained following the reaction and all primers are listed in Table 1. Three replications for each group were used for qPCR analysis. The Cq values (cycle threshold) of the gene were calculated using the 2^−ΔΔCt^ method. The formulas used was as follow: 2^−ΔΔCt^ = 2^−[(Cq of target gene−Cq of^ ^*RPS11*)experiment−(Cq of target gene−Cq of^ ^*RPS11*)control]^ (Livak and Schmittgen, 2001).

**TABLE 1:** Primers used in this study.

Primers	Sequence (5’→3’)
For dsRNA synthesis	
*GFP*-dsRNA-F	AAG​GGC​GAG​GAG​CTG​TTC​ACC​G
*GFP*-dsRNA-R	CTT​GAC​CTC​GGC​ACG​CGT​CTT​GT
*GFP*-dsRNA-T7-F	TAA​TAC​GAC​TCA​CTA​TAG​GGA​AGG​GCG​AGG​AGC​TGT​TCA​CCG
*GFP*-dsRNA-T7-R	TAA​TAC​GAC​TCA​CTA​TAG​GGC​TTG​ACC​TCG​GCA​CGC​GTC​TTG​T
*Akirin*-dsRNA-F	GTG​TTC​CAA​TGT​GCG​TTA​GTC
*Akirin*-dsRNA-R	GGCTGGTGCTGGTGATGT
*Akirin*-dsRNA-T7-F	TAA​TAC​GAC​TCA​CTA​TAG​GGG​TGT​TCC​AAT​GTG​CGT​TAG​TC
*Akirin*-dsRNA-T7-R	TAA​TAC​GAC​TCA​CTA​TAG​GGG​GCT​GGT​GCT​GGT​GAT​GT
For qPCR	
Q-*RPS11*-F	CCG​ATC​GTG​TGG​CGT​TGA​AGG​G
Q-*RPS11*-R	ATG​GCC​GAC​ATT​CTT​CCA​GGT​CC
Q-*Akirin*-F	GCC​CGA​AAA​TGA​CCC​CTG​AA
Q-*Akirin*-R	CCC​AAC​CTG​GCG​AAA​TGT​GA
Q-*TOR*-F	GGCTACAGGGATGTCAAA
Q-*TOR*-R	GAG​ATA​GAT​TCA​AAC​GGA​AAG
Q-*AMPK*-F	CCA​GAT​GCT​TCG​CTT​TAC​G
Q-*AMPK*-R	TTG​GCT​TTG​GTA​GGT​CGT​TA
Q-*Met*-F	AGT​GGC​AGC​GAG​CGA​TGA​TT
Q-*Met*-R	TGA​GGC​GCA​GCA​AAA​AGG​AG
Q-*Kr-h1*-F	TGA​TGA​GGC​ACA​CGA​TGA​CT
Q-*Kr-h1*-R	ATG​GAA​GGC​CAC​ATC​AAG​AG
Q-*InR*-F	GAG​TGC​AAC​CCG​GAG​TAT​GT
Q-*InR*-R	TCT​TGA​CGG​CAC​ACT​TCT​TG
Q-*IRS*-F	ATA​TCC​GCT​TCC​CCG​AGT​ACA
Q-*IRS*-R	TTT​GGT​TCA​GAA​TCT​CCG​TCC​T
Q-*PI3K*-F	TGT​TCG​TCC​CAG​CAG​TTC​G
Q-*PI3K*-R	TCG​GTG​GAG​GTT​GAG​ATT​G
Q-*VgR*-F	AGG​CAG​CCA​CAC​AGA​TAA​CCG​C
Q-*VgR*-R	AGC​CGC​TCG​CTC​CAG​AAC​ATT
Q-*Vg1*-F	CAG​TCC​CCA​AGT​GCC​AGA​AA
Q-*Vg1*-R	ATT​GGG​CAT​TCT​TGC​CGT​GA
Q-*Vg2*-F	TCTGCGCCATCGCGGTT
Q-*Vg2*-R	CCT​CCG​CTT​AGG​TTC​TGG​TG
Q-*Vg3*-F	GCG​GAA​ACG​GAC​CAT​GGA​A
Q-*Vg3*-R	CAG​GGG​CAA​CTG​CTT​GTA​CT
Q-*Vg4*-F	CCT​CCT​TCT​CCA​TTC​TCA​CCA​G
Q-*Vg4*-R	TGC​TTG​AAT​GCT​TCC​CAA​CTC

### 2.5 Organizational distribution detection

Female BPH individuals were dissected under a microscope on the first day of adulthood. Various fresh tissues (head, wing, cuticle, leg, ovary, fat body, and gut) were washed three times in 1× phosphate-buffered saline (PBS) (1.47 mM KH_2_PO_4_, 8.1 mM NaH_2_PO_4_, 137 mM NaCl, 2.68 mM KCl, and pH 7.4). The same organs from 30 to 80 adults were pooled followed by total RNA extraction and reverse-transcription. Subsequently, the expression distribution of *NlAkirin* in these organs was determined using qPCR.

### 2.6 RNA interference experiment

A plasmid containing gene fragments of *NlAkirin* and control *GFP* (GenBank ID: KU306402.1) was used as a template, in addition to primers containing the T7 promoter region 5′-TAT​ACG​ACT​CAC​TAT​AGG-3^,^ to perform the amplification reaction as follows: pre-denaturation at 95°C for 5 min, denaturation at 95°C for 30 s, annealing at 48°C for 30 s, extension at 72°C for 1 min with 35 cycles, and a further extension for 10 min at 72°C. PCR products, utilizing the T7 RiboMAX™ Express RNAi System (Promega, Madison, United States), were used to synthesize and purify a large amount of dsRNA according to the manufacturer’s instructions. A NanoDrop 2,000 spectrophotometer (Thermo, Waltham, United States) was used to measure the dsRNA purity (A260/A280) and yield, and dsRNA size was detected using 1% agarose gel electrophoresis.

Using a microinjection system (Eppendorf, Hamburg, Germany), ds*Akirin* or ds*GFP* was injected into the segments of one-day-old BPH adults (dose: 200 ng), and the adults were reared in glass feeding tubes containing rice seedlings. Knockdown efficiency was confirmed via qPCR at 24, 48, and 72 h following dsRNA injection.

### 2.7 Analysis of reproduction capacity of female BPHs

Each pair of dsRNA-injected females and untreated one-day-old male BPH adults was placed in a glass tube containing rice seedlings for cultivation. The rice stems were replaced every 2 days during the oviposition stage. After treatment with dsRNA, the number of eggs on the rice stem was counted. Observations were conducted continuously for 15 days under a Nikon SMZ1500 stereoscopic zoom microscope (Nikon, Tokyo, Japan). Hatching rate was monitored over an 18-day period. Eggs that remained unhatched after 18 days postoviposition were considered infertile. In addition, the BPH ovaries were dissected in 1 × PBS buffer under the microscope. Morphological changes were observed and photographed using NIS Elements BR software (Nikon, Tokyo, Japan).

### 2.8 JH III and Ecd content measurements

To understand the effect of *NlAkirin* on the JH and Ecd levels, we injected ds*Akirin* into one-day-old BPH adult females, collected unmated females at different times (24 and 48 h) after injection, and conducted enzyme-linked immunosorbent assay (ELISA). Female samples were collected, ground into a homogenate (sample: PBS = 1:9, m/m), and centrifuged at 3,000 rpm for 10 min; the supernatant was subsequently used to measure the JH III and Ecd contents. We used insect JH III (Enzyme Immune, Jiangsu, China) and Ecd (Enzyme Immune, Jiangsu, China) ELISA kits to measure the JH III and Ecd contents, respectively according to the manufacturer’s instructions, unless the concentrations of the standard substances differed; the same steps were used for determining the two hormones. The optical density (OD) value corresponding to each standard substance concentration at 450 nm was taken as the abscissa, and the standard substance concentration was taken as the ordinate to draw the standard curve. We inserted the sample OD value into the standard curve regression equation to calculate the sample concentration and multiplied it by the dilution factor to obtain the actual sample concentration. To understand the effect of mating on females, we mated female ds*Akirin-*treated BPH individuals with untreated males, placed them in pairs on rice, and collected them at two time points (24 and 48 h) after mating to measure the JH III and Ecd levels.

### 2.9 RNA sequencing analysis of BPHs after RNAi

To understand the effect of *NlAkirin* on the levels of other genes, we injected ds*Akirin* into one-day-old BPH adults. At 48 h after dsRNA injection, total RNA was extracted from the BPHs using TRIzol reagent (Invitrogen, Carlsbad, CA, United States). Three replicates were tested for each ds*Akirin* and ds*GFP* group; concentrations were measured, and the larvae were immediately placed on dry ice in preparation for RNA sequencing (RNA-seq). All the samples were sequenced with an Illumina NovaSeq 6000 (paired-end sequencing length, 150 bp) (Novogene, Beijing, China). The sequencing fragments were derived from image data obtained by high-throughput sequencers and transformed into sequence data through CASAVA base recognition. Fragments underwent crucial data filtration steps to ensure analytical quality and reliability, which involved removing reads with adapters, excluding those with ‘N' bases, and filtering out low-quality reads where bases with a Qphred score ≤5 constituted over 50% of the total read length. Additionally, assessments for the Q20, Q30, and GC contents were carried out on the refined data. Reads were mapped against the BPH genome database (RefSeq: GCF_000757685.1) using the hisat2 v2.0.5 program. Subsequently, the featureCounts software (1.5.0-p3) was used to quantify the reads mapped to individual genes. Furthermore, the fragments per kilobase million (FPKM) value for each gene was computed based on gene length. DESeq2 (version 1.20.0) was used to conduct differential expression analysis between two comparison groups. ClusterProfiler (version 3.8.1) was then applied to perform gene ontology (GO) enrichment analysis of differentially expressed genes and to statistically enrich genes in kyoto encyclopedia of genes and genomes (KEGG) pathways.

### 2.10 Detection and verification of reproduction-related pathway genes

According to the above RNAi method, at 24, 48, and 72 h after dsRNA injection, total RNA was extracted from five fresh female BPHs using the TRIzol reagent (Invitrogen, Carlsbad, CA, United States). Three replicates were tested for each of the ds*Akirin* and ds*GFP* groups, and the mRNA expressions of the reproduction-related *Vg*, *Vitellogenin receptor* (*VgR*), and TOR (*target of rapamycin*, *TOR*), (*AMP-activated protein kinase*, *AMPK*)), JH ((*Methoprene-tolerant*, *Met*), (*Kruppel-homolog 1*, *Kr-h1*)), and insulin ((*insulin receptor*, *InR*), (*insulin receptor substrate*, *IRS*), (*Phosphotidylinositol 3 kinase*, *PI3K*)) pathway-related genes were detected using qPCR (primers are listed in Table 1).

### 2.11 Ovarian protein content in female BPHs

According to the RNAi method described above, 50% of the dsRNA-treated females were kept in a normal culture, whereas the remaining 50% were paired with untreated males for one-to-one feeding. After 72 h, ovaries were dissected from unmated and mated females and lysed with 50 μL RIPA lysis buffer. Subsequently, 1 μL of 1% protease inhibitor mixture (Epizyme, Shanghai, China) was added, and the protein contents in the ovaries were detected using the standard curve method according to the instructions of the BCA protein quantification kit (Solarbio, Beijing, China).

### 2.12 Statistical analysis

Data are expressed as the mean ± standard error (SE) based on three independent biological replicates. Differences were considered statistically significant at *P* < 0.05. Data were analyzed using SPSS (version 29.0; SPSS Inc., Chicago, United States) and GraphPadPrism software (version9.0.0; GraphPad Software Inc., California, United States).

## 3 Results

### 3.1 Sequence and tissue expression profile analysis of NlAkirin

**FIGURE 1:**
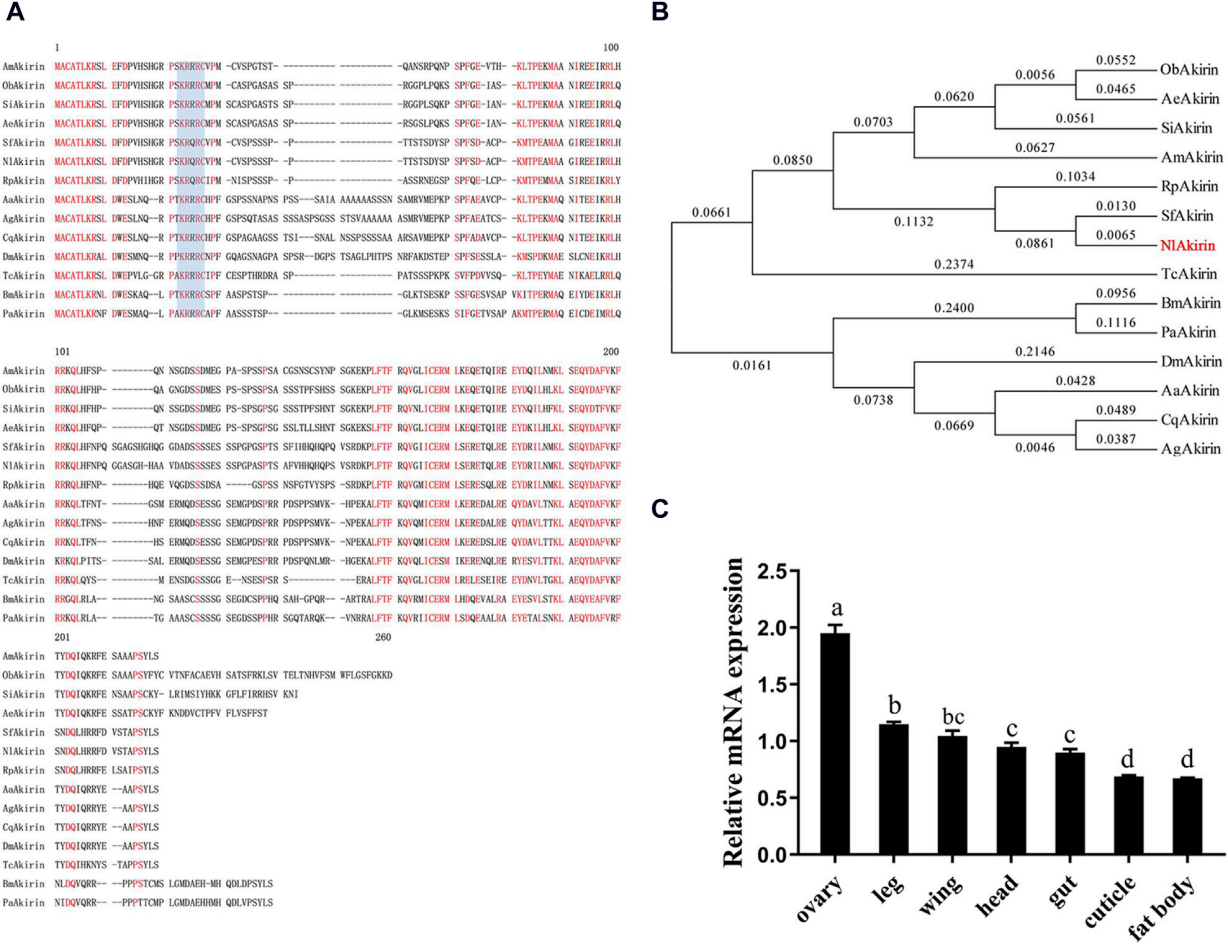
Sequence alignment and tissue expression profile analysis of insect Akirin. **(A)** Akirin protein sequence alignment for insects. The conserved nuclear localization signal (NLS) is shaded blue. **(B)** Phylogenetic tree of insect Akirin based on amino acid sequences. Phylogenetic tree constructed using the neighbor-joining method. The numbers on the branches of the phylogenetic tree represent bootstrap values from 1000 replicates, and the scale represents genetic distances. The red color represents NlAkirin from the BPH, and the GenBank IDs of each species are as follows: AmAkirin (*Apis mellifera*, XP_395252.2), SiAkirin (*Solenopsis invicta*, XP_011169583.1), AeAkirin (*Acromyrmex echinatior*, XP_011066641.1), SfAkirin (*Sogatella furcifera*, MG744348), NLAkirin (*Nilaparvata lugens*, AWT86615.1), RpAkirin (*Riptortus pedestris*, BAN21089), AgAkirin (*Anopheles gambiae*, XP_308938.4), CqAkirin (*Culex quinquefasciatus*, XP_001863200.1), AaAkirin (*Aedes albopictus*, ACF49499.1), DmAkirin (*Drosophila melanogaster*, NP_648113.1), TcAkirin (*Tribolium castaneum*, XP_971340), ObAkirin (*Ooceraea biroi*, EZA59122.1), BmAkirin (*Bombyx mori*, NP_001243977.1), and PaAkirin (*Pararge aegeria*, JAA80553.1). **(C)** Expression levels of *NlAkirin* in different tissues of BPH females. Statistical analysis was performed via multiple comparison. Data are shown as mean ± *SE* of three independent experiments. a, b, c, and d: Turkey test, *P* < 0.05. The presence of identical letters indicates no significant difference between these two sets of data.

The *NlAkirin* (GenBank ID: AWT86615.1) cDNA contained a 585 bp open reading frame that encoded 194 amino acids. The predicted molecular weight and theoretical pI were approximately 21.54 kDa and 8.66, respectively. The N-terminus of the NlAkirin protein did not contain a signal peptide but displayed a typical nuclear localization signal (KRQRC/KRRRC) located at the 23rd–27th amino acids of the N-terminus (Figure 1A). A multi-sequence alignment of the NlAkirin amino acid sequences of 14 insects revealed that individuals belonging to the Hymenoptera, Hemiptera, Diptera, Coleoptera, and Lepidoptera orders had conserved sequences at both the N- and C-termini.

Our constructed phylogenetic evolutionary tree revealed that Akirin in insects was divided into two branches: the first consisting of Diptera and Lepidoptera, and the second comprising Coleoptera, Hemiptera, and Hymenoptera (Figure 1B). NlAkirin was closely related to *S*. *furcifera*, which belongs to the family Delphacidae.

As shown in Figure 1C, qPCR analysis revealed the expression of *NlAkirin* across all seven tissues (head, wing, cuticle, leg, ovary, fat body, and gut). Notably, the expression level was highest in the ovary, whereas the lowest expression was observed in the fat body and cuticle. These findings suggest that *NlAkirin* may influence the reproductive regulation of BPH females.

### 3.2 *NlAkirin* reduces reproductive capacity of female BPHs

**FIGURE 2:**
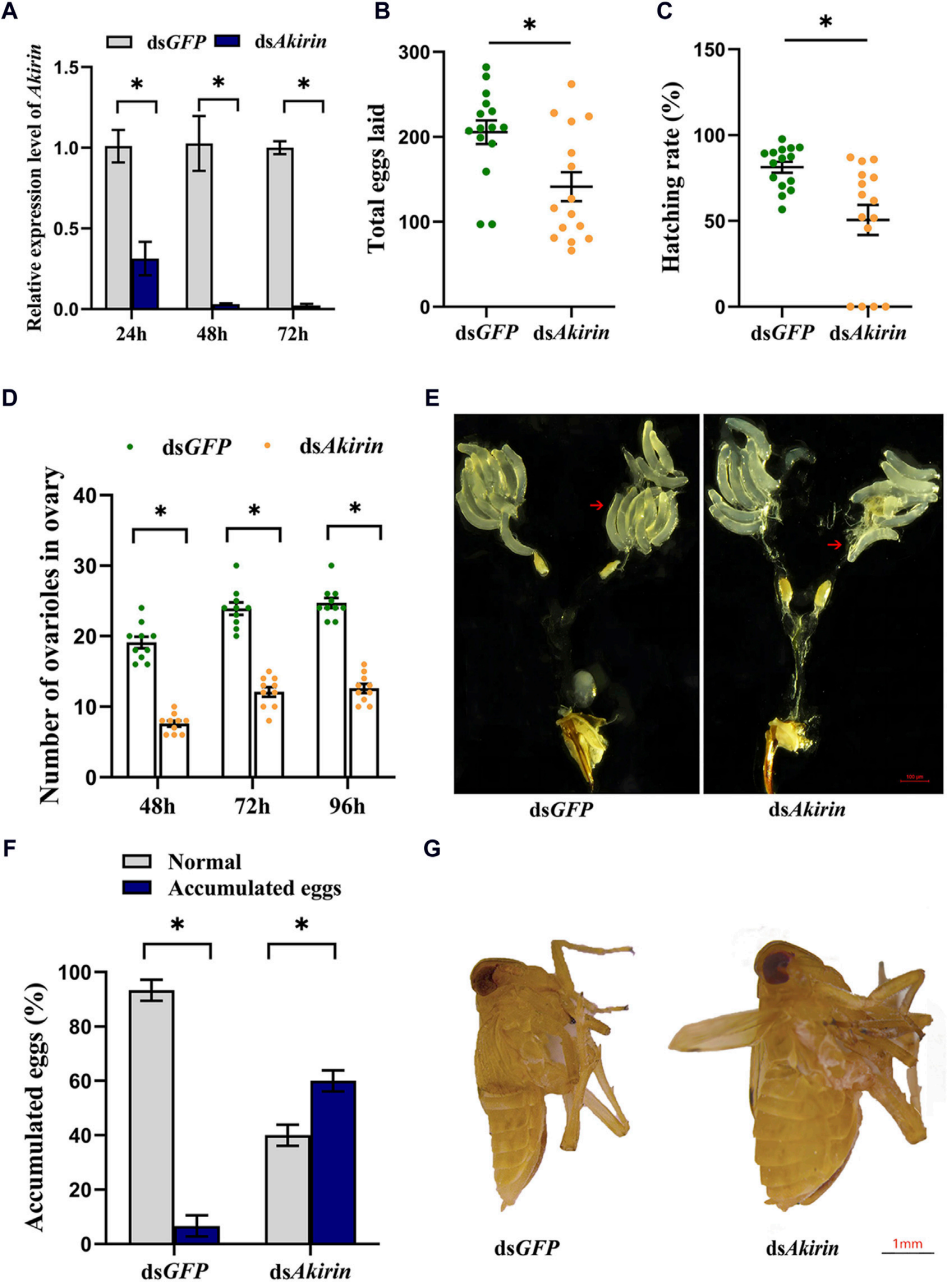
Effect of *NIAkirin* on the reproduction of BPH females. **(A)** mRNA expression level of *NIAkirin* after RNAi. **(B)** Number of total eggs laid. **(C)** Hatching rate of eggs. **(D)** Number of ovarioles in the ovary. **(E)** Representative images of the number of ovarioles, scale bar represents 100 μm. **(F)** Proportion of mortality due to the large accumulation of eggs in abdomens. **(G)** Representative images of accumulation of eggs in abdomens, scale bar represents 1 mm. Statistical analysis was performed via the *t*-test. Data are shown as the mean ± *SE* of three independent experiments. *P < 0.05; ns, non-significant difference.

A substantial interference effect was observed following the ds*Akirin* injection across all three time points. As shown in Figure 2A, compared with the control group injected with ds*GFP*, the expression of *NlAkirin* in females experienced significant downregulation (*P* < 0.05) from 24 to 72 h post-ds*Akirin* injection, with a downregulation range of approximately 69%–98%. These results underscore the effective silencing of *NlAkirin* expression through the microinjection method.

Regarding the knockdown of *NlAkirin* expression in females, the average number of eggs laid by adult females in the ds*Akirin* treatment group was lower than that in the ds*GFP* treatment group (Figure 2B). In addition, the egg-hatching rate in the ds*Akirin*-treated group was significantly lower than that in the ds*GFP* group (51% and 81%, respectively; Figure 2C). The number of ovarioles in individuals following ds*Akirin* injection was significantly lower than that in individuals belonging to the control ds*GFP* group (Figures 2D, E). No significant difference in mortality was observed between the ds*GFP* and ds*Akirin* groups following dsRNA injection; however, approximately 60% of females that died in the ds*Akirin* treatment group showed a large accumulation of eggs in their abdomens (Figures 2F, G).

### 3.3 *NlAkirin* regulates the expression of genes related to reproductive capacity

Total RNA extraction from the ds*Akirin* and ds*GFP* groups was relatively complete, meeting the sequencing quality requirements for total RNA samples to establish a transcriptome library. The raw read count results of RNA-seq have been uploaded to the National Center for Biotechnology Information (NCBI) database (BioProject accession: PRJNA1075481). The statistical power of this experimental design, calculated in RNASeqPower, was 0.99; the sequencing depth was 60.16 (Supplementary Table S1); the percentages of Q30 and GC bases in the six quality control sequences (clean data) were ≥92.1 and ≥34.45%, respectively; and the overall sequencing error rate was 0.33% (Supplementary Table S2). The results indicate that the transcriptome sequencing results were satisfactory, providing useful raw data for subsequent data assembly. In addition, Pearson’s correlation coefficient analysis of the gene expression levels between the sample group showed an *R* ^2^ value of >0.86; therefore, the gene expression profiles of the biological replicate samples were consistent and differed between samples, and the experimental data were reliable (Supplementary Figure S1).

**FIGURE 3:**
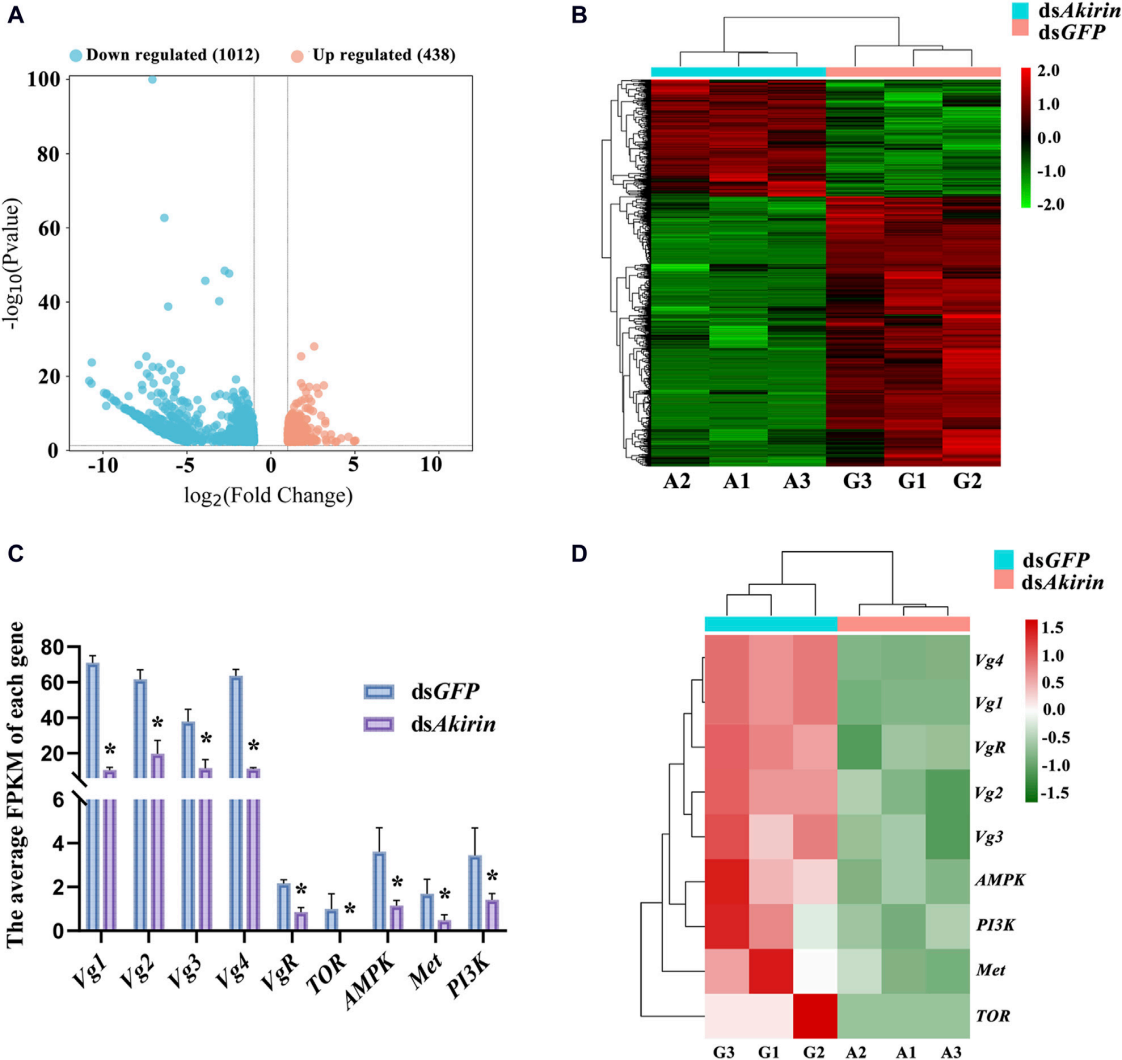
Analysis of RNA-seq results after ds*Akirin* injection. **(A)** Volcano plot of DEGs. **(B)** Cluster heatmap of DEGs. G1–G3, the three replicates of ds*GFP* treatment; A1–A3, the three replicates of ds*Akirin* treatment; The left side depicts the clustering of different genes. The number displayed on the right indicates the level of gene expression. **(C)** The average FPKM of each gene. The gene ID numbers for *Vg1* to *Vg4* were 111061268, 111057493, 111061289, and 111061279, respectively. * padj≤0.05 and |log2FoldChange|≥1.0. **(D)** Cluster heatmap of genes related to female insect reproduction in transcriptome sequencing results; The left side depicts the clustering of different genes. The number displayed on the right indicates the level of gene expression.

To identify *Akirin*-related genes, we evaluated the gene expression levels of each unigene. Cluster heatmaps of the differentially expressed genes (DEGs) are shown in Figures 3A, B. There were 438 upregulated and 1,012 downregulated genes in the ds*Akirin* group compared with those in the ds*GFP* control group (Supplementary Table S3). As shown in Supplementary Figure S2, GO functional enrichment analysis of the DEGs was used to select the top 10 most significant GO functions from biological process (BP), cell component (CC), and molecular function (MF). KEGG pathway enrichment analysis was performed on the selected DEGs. The important pathways are shown in Supplementary Figure S3.

Moreover, the transcriptome sequencing results demonstrated that *NlAkirin* downregulation led to a significant decrease in *Vg* (Gene IDs 111061279, 111061289, 111061268, and 111057493) and *VgR* (Gene ID 111052579) expression, both of which were related to female insect reproduction relative to the ds*GFP* group. Owing to the presence of four *Vg* gene ID numbers in the transcriptome sequencing data, we aligned the nucleotide ( Supplementary Figure S4) and amino acid ( Supplementary Figure S5) sequences of the four *Vg* genes. The sequences of these four *Vg* genes mainly differed at the 3^,^ end and showed significant differences in FPKM values from the ds*GFP* group in transcriptome data. Additionally, the FPKM values of genes related to Vg synthesis, including the *TOR* (Gene ID 111045108) and *AMPK* (Gene ID 111061083) genes in the TOR pathway, *Met* (Gene ID 111058432) gene in the JH pathway, and *PI3K* (Gene ID 111047698) gene in the insulin signaling pathway were significantly downregulated (Figures 3C, D).

### 3.4 Verification of the impact of decreased *NlAkirin* expression on mRNA levels of *Vg*, *VgR*, and associated pathway genes

*NlAkirin* downregulation significantly affects the expression levels of *Vg*, *VgR*, and four TOR/JH/insulin signaling pathway genes at the mRNA level (Figure 3C). However, certain genes in the pathway did not exhibit significant differences with padj ≤0.05 and |log2FoldChange| ≥ 1.0 due to the limited depth of transcriptome sequencing. Hence, we identified some genes from these pathways and conducted qPCR validation collectively.

**FIGURE 4:**
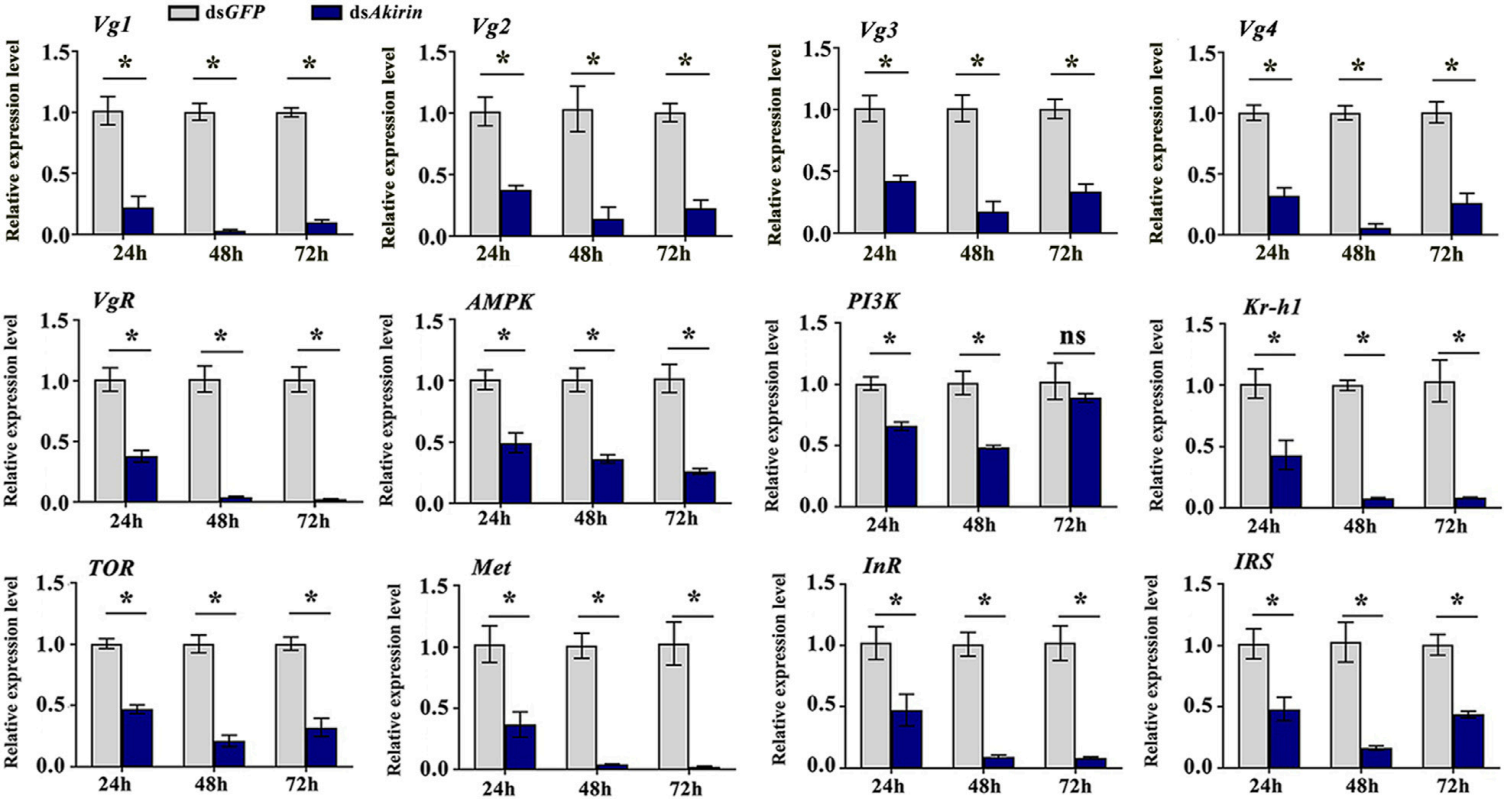
mRNA expression of *Vg*, *VgR*, and related pathway genes. Statistical analysis was performed using the *t*-test. Data are shown as the mean ± *SE* of three independent experiments. The gene ID numbers for *Vg1* to *Vg4* were 111061268, 111057493, 111061289, and 111061279, respectively. *P < 0.05; ns, non-significant difference.

The qPCR results confirm that mRNA expression of the *Vg* and *VgR* genes at 24, 48, and 72 h in the ds*Akirin* treatment group exhibited substantial downregulation compared with those in the ds*GFP* control group, showing notable reductions of approximately 62%–92% and 63%–98%, respectively (Figure 4). The mRNA expression of *InR* (Gene ID 111056107), *IRS* (Gene ID 111053511) and *PI3K* genes in the insulin signaling pathway was significantly downregulated by approximately 52%–91%, 52%–83%, and 10%–41%, respectively; that of the *Met* and *Kr-h1* (Gene ID 111055513) genes in the JH pathway was downregulated by approximately 63%–97% and 57%–92%, respectively, and that of the *TOR* and *AMPK* genes in the TOR pathway was downregulated by approximately 53%–79% and 50%–74%, respectively.

### 3.5 *NlAkirin* regulates the titers of JH III and Ecd

**FIGURE 5:**
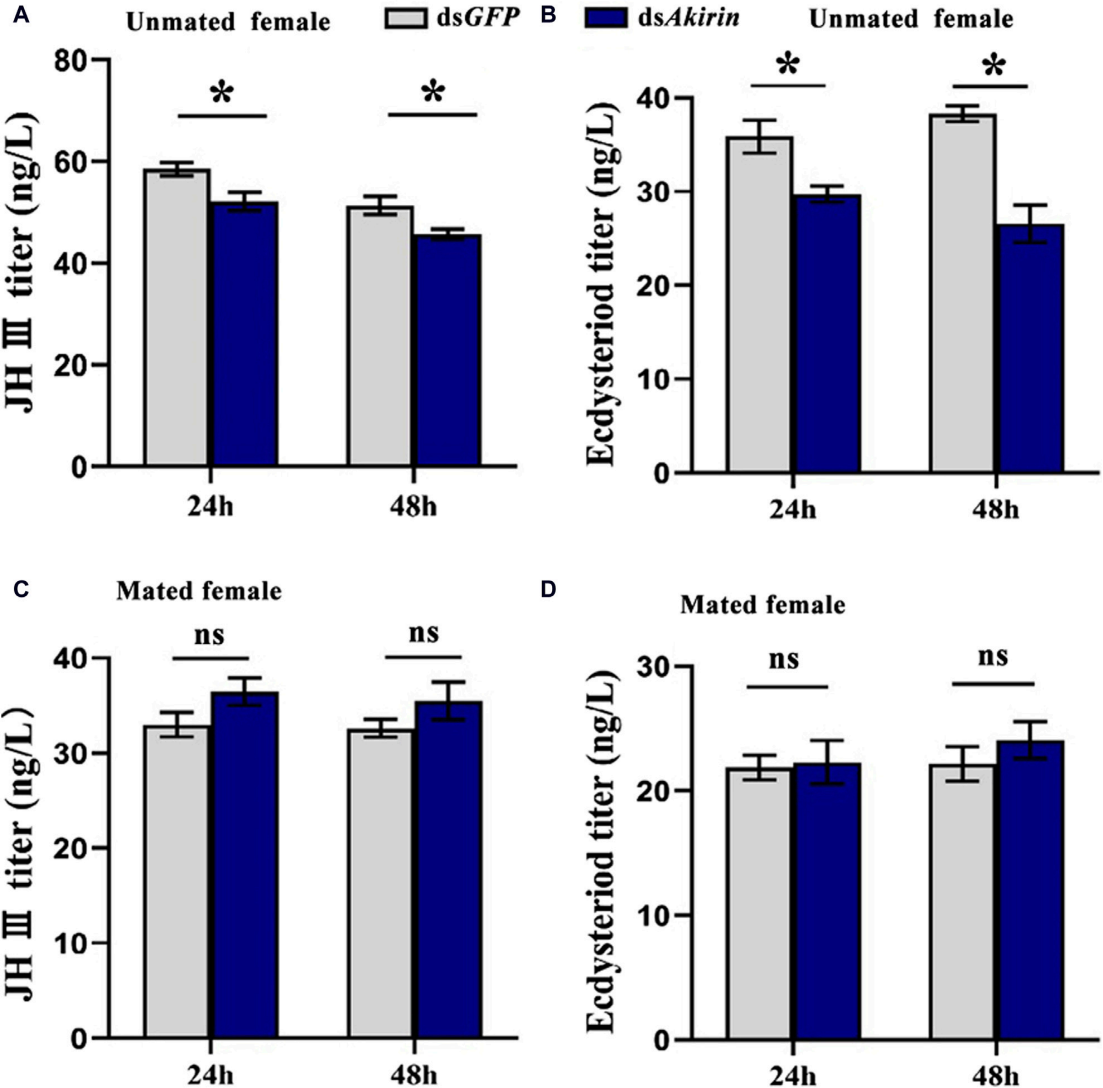
Titer results for levels of JH III and Ecd in BPH females treated with ds*Akirin*. **(A)** JH III titer in unmated females following ds*Akirin* injection. **(B)** Ecd titer in unmated females after ds*Akirin* injection. **(C)** JH III titer in mated females after ds*Akirin* injection and mating with untreated males. **(D)** Ecd titer in mated females after ds*Akirin* injection and mating with untreated males. Statistical analysis was performed using the *t*-test. Data are shown as the mean ± *SE* of three independent experiments. *P < 0.05; ns, non-significant difference.

In unmated females, the titers of JH III or Ecd were significantly lower in the ds*Akirin*-treated group than those in the ds*GFP* control group (Figures 5A, B). To understand the effect of mating behavior on females, we mated each single female BPH treated with ds*Akirin* with an untreated male BPH, placed them in pairs on rice, and collected the females at different time points after mating for JH III and Ecd titer determination. As shown in Figures 5C, D, compared with the mated females in the ds*GFP* control group, the JH III and Ecd titers of mated females treated with ds*Akirin* returned to normal levels, with no significant difference compared with the ds*GFP* group.

### 3.6 *NlAkirin* modulates the levels of ovarian proteins

**FIGURE 6:**
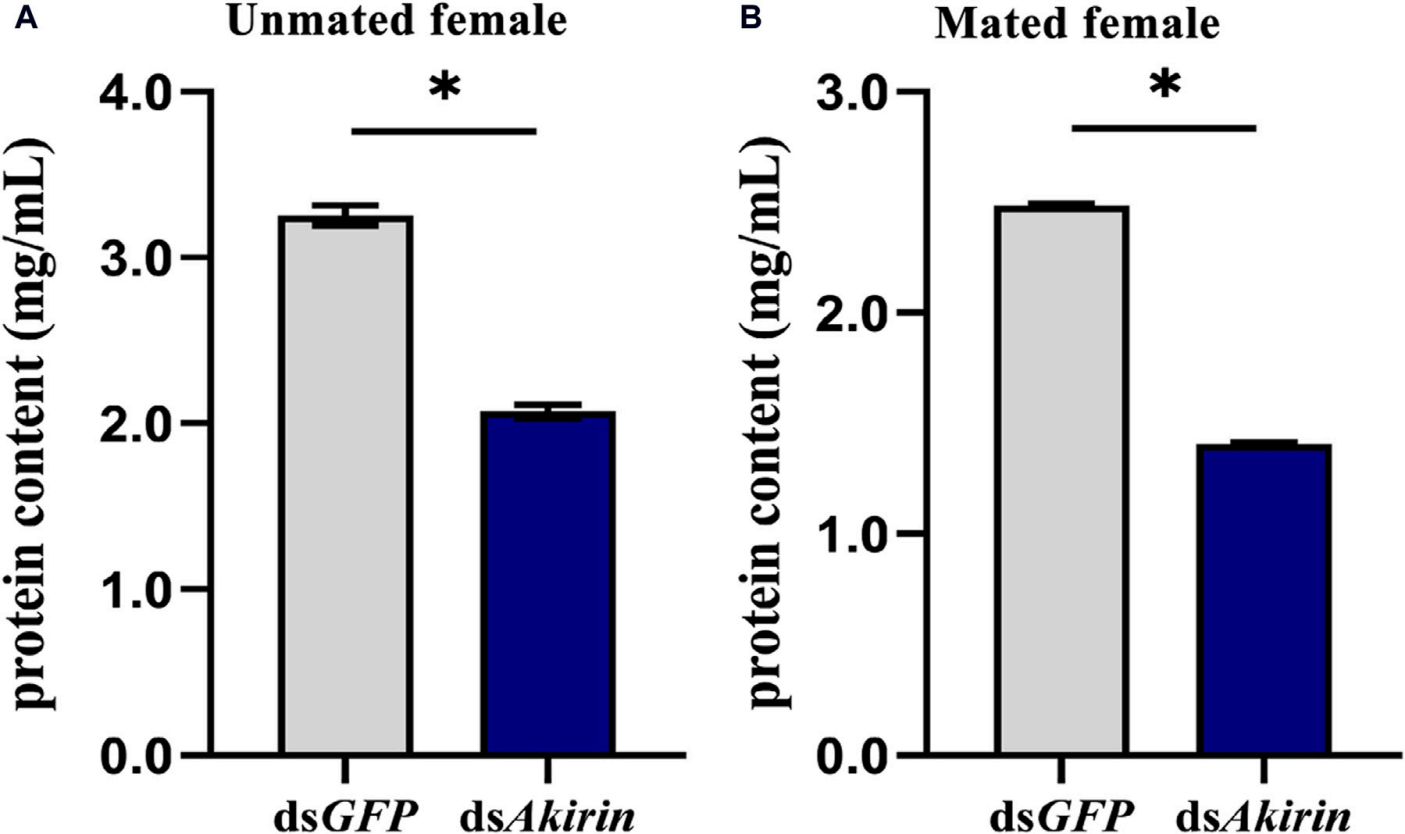
Protein content in the ovary of BPH females treated with ds*Akirin*. **(A)** Ovarian protein content in unmated females following injection of ds*Akirin*. **(B)** Ovarian protein content in females after mating with untreated males after injection of ds*Akirin*. Statistical analysis was performed using a *t*-test. Data are shown as mean ± *SE* of three independent experiments. *P < 0.05.

Compared with that in the ds*GFP* control group, the ovarian protein content in the ds*Akirin*-treated group was significantly lower in unmated female BPHs (Figure 6A). As shown in Figure 6B, compared with that in the ds*GFP* control group, the ovarian protein content in ds*Akirin*-treated mated females decreased, with significant differences compared with the ds*GFP* group.

## 4 Discussion

In this study, we investigated the role of *NlAkirin* in female BPH reproduction. Amino acid sequence alignment results showed that NlAkirin from different insect orders had a high homology and contained highly conserved amino acid sequences in different regions. Additionally, Akirin contained an NLS in the N-terminal region, indicating that the protein influences the nucleus. The analysis of the *NlAkirin* expression characteristics in different BPH tissues showed that although *NlAkirin* mRNA was detected in all tissue types, its expression varied significantly. *NlAkirin* had the highest expression level in the ovary; this was consistent with the results for *S*. *furcifera* (Chen et al., 2018) and *C*. *elegans* (Polanowska et al., 2018). The tissue-specific distribution of a gene generally correlates closely with its function. Thus, we speculate that Akirin is intricately linked to the reproductive processes of BPH.

To further investigate the effect of *NlAkirin* on the reproduction capacity of female BPH, we used RNAi injection to interfere with the expression of *NlAkirin* in one-day-old female BPH individuals, and the total number of eggs laid and hatching rate of individual females were detected. After mating with untreated males, females treated with ds*Akirin* showed reduced egg production and a lower egg hatching rate in the pregnant state, indicating that *NlAkirin* affects the reproductive capacity of female BPHs. Similar results have been reported for other insects, ticks, and mites (Busby et al., 2012; Krossa et al., 2015; Tartey et al., 2015). For example, in *A*. *arabiensis*, the expression of Akirin was inhibited by siRNA and the reproductive capacity and fertility decreased by 25% and 29%, respectively (Letinic et al., 2020). Similarly, the survival rate decreased by 14%, and egg production decreased by 52% (Letinic et al., 2020) compared with those of the control in *A*. *arabiensis*. Moreover, the efficacy of three antigens–recombinant Akirin from *A*. *arabiensis*, *Aedes albopictus*, and Q38 (Akirin/Subolesin chimera)–was evaluated for novel vector control of *A*. *arabiensis*. Results showed that all three antigens successfully reduced *A*. *arabiensis* reproductive capacities (Letinic et al., 2021). In *D*. *gallinae*, the efficacy of vaccination with Akirin for controlling mite oviposition was demonstrated by a 42% reduction after feeding on vaccinated hens (Lima-Barbero et al., 2019).

To clarify whether *NlAkirin* affects the expression of reproduction-related genes in female BPH at the molecular level, we performed transcriptome sequencing on the BPH individuals injected with ds*Akirin*. The results showed that interfering with the expression of the *NlAkirin* gene significantly downregulated the mRNA expression of *Vg* and *VgR*, which are closely related to female insect fertility and served as a molecular marker for the reproductive capacity of BPHs (Qiu et al., 2016; Dong et al., 2021). Simultaneously, *NlAkirin* interference also significantly reduced the mRNA expression levels of genes related to the TOR, JH, and insulin signaling pathways. This finding confirmed that *NlAkirin* regulates the reproduction capacity of female BPHs by inhibiting Vg synthesis at the mRNA level.

As nutrient sensors, the TOR and insulin signaling pathways participate in the biosynthesis of JH and Ecd and collaborate with these components to regulate insect yolk production (Smykal and Raikhel, 2015; Roy et al., 2018). For example, in *Aedes aegypti* and *N*. *lugens*, the expression of *Vg* can be significantly inhibited by reducing TOR activity through a rapamycin treatment or RNAi technology (Lu et al., 2016). In addition, TOR can positively elevate the biosynthesis of JH and yolk production. In *Tribolium castaneum*, *Blattella germanica*, and *Periplaneta americana*, the TOR and insulin signaling can enhance the expressions of *JH methyltransferase* (*Jhamt*), *Met* gene, and JH early response factor *Kr-h1* gene, and further stimulate the expression of Vg and the maturation of oocytes (Abrisqueta et al., 2014; Zhu et al., 2020). In *D*. *melanogaster* and *A*. *aegypti*, insulin and TOR signaling pathways also affect the synthesis and secretion of Ecd, which further affect the production and oviposition of the yolk (Tu et al., 2002; Dhara et al., 2013). In insects, ovarian development, egg formation, and ovulation are regulated by multiple signaling molecules, such as Ecd, JH, and matrix metalloproteinases (Monastirioti, 2003; Deady et al., 2015; Knapp and Sun, 2017; Luo et al., 2021). Insect metamorphosis is mainly controlled by the coordination of JH and Ecd. Ecd induces insect ecdysis and metamorphosis, whereas JH prevents 20E-induced metamorphosis, thereby maintaining a larval state. During the final larval stage, the JH titer significantly decreased or disappeared, resulting in the complete metamorphosis of insects from the larval stage to the pupal stage. In BPH individuals, JH regulates the expression of *Vg*, and silencing *NlVgR* significantly inhibits ovarian development and the accumulation of the Vg protein in the blood (Tufail et al., 2010). Some studies have reported that RNAi-*SPF-L* indirectly affects the JH III titer, leading to a downregulation of *Vg* expression and Vg protein synthesis and an inhibition of ovarian development and fertility (Ge et al., 2019).

To determine whether the effect of *NlAkirin* on the reproduction of females can be achieved through the regulation of JH and Ecd, we detected the titers of JH III and Ecd in females after injecting ds*Akirin*. The results showed that, compared with those in the ds*GFP* control group, the titers of JH III and Ecd in the ds*Akirin*-treated group of unmated females were significantly downregulated, whereas those in the ds*Akirin*-treated mated females did not differ from those of the control. Previous research has shown that mating behavior has a significant impact on female insects, especially in certain species, where it leads to rejection of subsequent males and promotes feeding, ovulation, oviposition, and longevity (Avila et al., 2011). Moreover, seminal fluids have complex compositions, including multiple amino acids, proteins, lipids, carbohydrates and hormones, such as JH or ecdysteroids (Wolfner, 1997). In some species, such as *Hyalophora cecropia* and *Heliothis species*, males transfer JH to females during mating, which results in increased JH titers in the female hemolymph (Wolfner, 1997; Srinivasan et al., 1998). In BPH, mating can stimulate the fertility of female individuals (Wang et al., 2010) and significantly increase the JH III titer and the *Vg* mRNA and protein levels (Ge et al., 2010; Lu et al., 2015). Therefore, we speculate that during the reproductive process, JH III and Ecd in semen are also partially transferred to the female BPHs, leading to an increase in the titers of JH III and Ecd after mating. Furthermore, by knocking down *juvenile hormone acid methyl transferase* (*Jhamt*)–the enzyme that converts JH acid to JH–preventing JH production in *B*. *mori* embryos results in only a slight delay in embryogenesis (Daimon et al., 2015). In our experimental results, the timing of the dsRNA experiment on female BPHs was relatively late, thus suggesting that the injection of ds*Akirin* might not have a significant impact on their survival rate.

The protein content of an insect’s ovaries has a direct impact on their reproductive capacity and is crucial for the development and maturation of eggs. We also observed that the total protein content of the ovary of female BPH before and after mating significantly decreased after injection with ds*Akirin* and did not recover due to mating behavior. The synthesis of insect Vg is a complex process regulated by various factors. In addition to hormones, it is also subject to the modulation of multiple signaling pathways (Parthasarathy and Palli, 2011; Abrisqueta et al., 2014; Wu et al., 2020; Zhu et al., 2020). In our study, downregulating *Akirin* expression in BPH revealed a sustained high interference efficiency, even after 72 h of interference, leading to consistently lower mRNA levels of *Vg* and *VgR*, and reduced protein synthesis levels compared with the control, even upon hormone recovery. Furthermore, reducing *Akirin* expression resulted in a significant decrease in the number of ovarioles and eggs in BPH. Consequently, the accumulation of proteins in the eggs was also notably reduced compared with the control ds*GFP* group, both before and after mating.

This study has shown the influence of NlAkirin on the reproduction of BPHs, yet several crucial mechanistic aspects remain unexplored. Notably, despite establishing a correlation between NlAkirin and the biosynthesis of Vg, along with its affiliated gene networks, the precise molecular mechanisms underlying this relationship remain elusive. Given that Akirin possesses the ability to modulate the transcriptional activity of regulated genes via its intricate interactions with DNA-binding proteins or transcription factors within the nucleus (Goto et al., 2008), we postulate that NlAkirin’s effect on female BPH reproduction is likely mediated through these complex protein-protein interactions. Future research endeavors will focus on identifying NlAkirin’s interacting proteins and delving deeper into the intricate molecular pathways that govern how NlAkirin regulates the reproductive capacity of female BPHs. Moreover, the ELISA method we used to determine the content of JH III and Ecd can be incorporated into future research by high performance liquid chromatography (HPLC) method to further validate and expand our findings.

## 5 Conclusion

Our results suggest that *NlAkirin* affects female BPH reproduction by regulating the mRNA expression of genes related to the *Vg*, *VgR*, TOR, JH, and insulin signaling pathways, in addition to the titers of JH III and Ecd. While previous studies have demonstrated the impact of Akirin on the reproductive capacity of female insects, to our knowledge, our study is the first to establish a connection between Akirin, Vg synthesis, and associated gene networks. The findings of this research provide novel insights into the regulatory role of Akirin in insect reproductive capacity, however, the molecular mechanism of how Akirin regulates BPH reproduction requires further research.

## Data Availability

The original contributions presented in the study are included in the article/Supplementary Material, further inquiries can be directed to the corresponding author.
