# Supplementary material for: Role of nuclear protein Akirin in the modulation of female reproduction in Nilaparvata lugens (Hemiptera: Delphacidae)
Source: Front Physiol. 2024 Jul 9;15:1415746. doi: 10.3389/fphys.2024.1415746 (PMC11264338; doi:10.3389/fphys.2024.1415746)
Supplement: Supplementary file 1 [file DataSheet2.PDF]

| Gene_ID | 1 ... 100 |
| --- | --- |
| 111061268 | MQGLTLLVAV LAVSGVSAS- -GPWNSNQQY RYHVQGRSLS AMHQSGSNQY VGMHLRAELE VEAKNENQAV FKISKAEYAD VHQNLSGGWQ QELRSNELQY |
| 111061279 | MNGLTLLLCA IAVAGVSASG SGPWNSNQQY RYHVQGRSLS AMHQSGSNQY VGMHLRAELE VEAKNENQAV FKISKAEYAD VHQNLSGGWQ QELRSNELQY |
| 111057493 | MNGLTLLLCA IAVAGVSASS SGPWNSNQQY RYHVQGRSLS AMHQSGSNQY VGMHLRAELE VEAKNENQAV FKISKAEYAD VHQNLSGGWQ QELRSNELQY |
| 111061289 | MKGITLIFCV IAVAGVSASG NGPWNSNQQY RYHVQGRSLS AMHQSGSNQY VGMHLRAELE VEAKNENQAV FKISKAEYAD VHQNLSGGWQ QELSSNELQY |
| | 101 ... 200 |
| 111061268 | KQLPLSQANQ VFQVNYKQGA VRSLQVNRNT PTWELNMIKG FVSLFQVDVM AXNAIKSRRN IVPNGQQVSG SFKVMEDSVT GKCETHYDVD ELPMRVVQQH |
| 111061279 | KQLPLSQANQ VFQVNYKQGA VRSLQVNRNT PTWELNMIKG FVSLFQVDVT GQNAIKSRRN IVPNGQQVSG SFKVMEDSVT GKCETHYDVD ELPMRVVQQH |
| 111057493 | KQLPLSQANQ VFQVNYKQGA VRSLQVNRNT PTWELNMIKG FVSLFQVDVT GQNAIKSRRN IVPNGQQVSG SFKVMEDSVT GKCETHYDVD ELPMRVVQQH |
| 111061289 | KQLPLSQANQ VFQVNYKQGA VRSLQVNRNT PTWELNMIKG FVSLFQVDVT GQNAIKSRRN IVPNGQQVSG SFKVMEDSVT GKCETHYDVD ELPMRVVQQH |
| | 201 ... 300 |
| 111061268 | PEIAPLAVKQ QGQGQGQGQS HSRLIQVVKS RNFSNCDNPV TYHFGFTQET NFEPASNQMG NLVSRAAMGH MIIAGEIESF TIHSSVTQNE IAISPFGYNQ |
| 111061279 | PEIAPLAVKQ QGQGQGQ--S HSRLIQVVKS RNFSNCDNPV TYHFGFTQET NFEPASNQMG NLVSRAAMGH MIIAGEIDSF TIHSSVTQNE IAISPFGYNQ |
| 111057493 | PEIAPLAVKQ QGQGQGQ--S HSRLIQVVKS RNFSNCDNPV TYHFGFTQES NFEPASNQMG NLVSRAAMGH MIIAGEIESF TIHSSVTQNE IAISPFGYNQ |
| 111061289 | PEIAPLAVKQ QGQGQGQ--S HSRLIQVVKS RNFSNCDNPV TYHFGFTQES NFEPASNQMG NLVSRATTSR ILLAGEPDSY TIHSSVTQNE IAISPFGYNQ |
| | 301 ... 400 |
| 111061268 | QKGVVGTLMN ATLVSVSHAS SGSPQSVQNP QKINDLVYEF NPASNSESNQ RSSHYTRQQA DNEDDSSSSS SSSDSSSSSS SSSSSSSSSS SSSEENNKNS |
| 111061279 | QKGVVGTLMN ATLVSVSHAS SGSPQSVQNP QKINDLVYEF NPASNSESNQ RSSHYTRQQA DNEDDSSSSS SSSDSSSSSS SSSSSSSSSS SSSEENNKNS |
| 111057493 | QKGVVGTLMN ATLVSVSHAS SGSPQSVQNP QKINDLVYEF NPASNSESNQ RSSHYTRQQA DNEDDSSSSS SSSDSSSSSS SSSSSSSSSS SSSEENNKNS |
| 111061289 | QKGVVGTLMN ATLVSVSHAS SGSPQSVQNP QKINDLVYEF NPASNSESNQ RSSHYTRQQA DNEDDSSSSS SSSDSSSSSS SSSSSSSSSS SSSEENNKSS |
| | 401 ... 500 |
| 111061268 | KKNN-----N NKNWNNKNQK KNNNNNRNNH NDNDNNQDNS NENNNDDAYW RSQQKTKSRS RRSILRNYNN ENDDDNNQNR NQNRNNNNNN N--DSSEESN |
| 111061279 | KKNNKNWNNN NKNWNNKNQK NNNNNNRNNH NDSDNNRDNS NENNNDDAYW RSQQKTKSRS RRSILRNYNN ENDYDNNQNR NQNRNNNNNN NNNDSSEESN |
| 111057493 | KKNN-----N NKNWNNKNQK KKNNNNRNNH NDNDNNQDNS NENNNDDAYW RSQQKTKSRS RRSILRNYNN ENDDDNNQNR NQNRNNNNNN NN-DSSEESN |
| 111061289 | KKNN-----I NKNWNNKNQN KNNNNNRNNH NNNDNNQDNS NENNNDDAYW RSQQKTKSRS RRSILRNYNN ENDDDHNQNR NQNRNNNNNN N--DSSEESN |
| | 501 ... 600 |
| 111061268 | ENQNNNK--- NWNNNNNNNN KNWNNNDN-K NWNNNDNN-- -----NKNRN NNDNDDSSSS SSSSSSSSSS SSSSSSSSSS SSDLDSSEEN WQQKPGMNDA |
| 111061279 | ENQNNN---- -KNWNNNNNN KNWNNNDN-K NWNNNDNN-- -----NKNRN NNNNDDSSSS SSSSSSSSSS SSSSSSSSSS SSDLDSSEEN WQQKPGMNDA |
| 111057493 | ENQNNNNKHN NKNWNNNDNN KNWNNNDNNK NWNNN----- -----NKNRN NNDNDDSSSS SSSSSSSSSS SSSSSSSSSS S-DLDSSEEN WQQKPGMNDA |
| 111061289 | ENQNNNNKHN NKNWNNNDNN KNWNNNDNNK NWNNNDNNKN WNNNNNKNRN NNDNDDSSSS SSSSSSSSSS SSSSSSSSSS SSDLDSSEEN WQQKPGMNDA |
| | 601 ... 700 |
| 111061268 | PRTPFLPHFV GVRGNSIQAD KQVDIVNEVQ KVAMRIGAQV QRPSAIPGQN TLTSFTILTR MIQTMSAKQI QEVKQRLFID RNNANGKSSA DAKKLQSWEA |
| 111061279 | PRTPFLPHFV GVRGNSIQAD KQVDIVNEVQ KVAMRIGAQV QRPSAIPGQN TLTSFSILTR MIQTLSAKQI QEVKQRLFID RNNANGKSSA DAKKLQSWEA |
| 111057493 | PRTPFLPHFV GVRGNSIQAD KQVDIVNEVQ KVAMRIGAQV QRPSAIPGQN TLTSFTILTR MIQTMSAKQI QEVKQRLFID RNNANGKSSA DAKKLQSWEA |
| 111061289 | PRTPFLPHFV GVRGNSIQAD KQVDIVNEVQ KVAMRIGAQV QRPSAIPGQN TLTSFTILTR MIQTMSAKQI QEVKQRLFID RNNANGKSSA DAKKLQSWEA |
| | 701 ... 800 |
| 111061268 | FKHATANAGT GPALEAIKNW VEKGDVRNEK AAELVAVLPR TARLPTDQYI KTFFQFATSS NVQNQKYLNS TIILGFSEIL RKAQVDSDTK HMRFGVHSFG |
| 111061279 | FKHATANAGT GPALEAIKNW VEKGDVRNEK AAELVAVLPR TARLPTDQYI KTFFQFATSS NVQNQKYLNS TIILGFSEIL RKAQVDSDTK HMRFGVHSFG |
| 111057493 | FKHATANAGT GPALEAIKNW VEKGDVRNEK AAELVAVLPR TARLPTDQYI KTFFQFATSS NVQNQKYLNS TIILGFSEIL RKAQVDSDTK HMRFGVHSFG |
| 111061289 | FKHATANAGT GPALEAIKNW VEKGDVRNEK AAELVAVLPR TARLPTDQYI KTFFQFATSS NVQNQKYLNS TIILGFSEIL RKAQVDSDTK HMRFGVHSFG |
| | 801 ... 900 |
| 111061268 | HLTSKHDQSL HQEYMPYLEE KLKSAFEKGD SQKIIVYIQA LGNTAHPRLL KTFEPYLEGK KSASRFQRLL MVASLYQMTR VHPTTARAVL YRIYKNPGEA |
| 111061279 | HLTSKHDQSL XQEYMPYLEE KLKSAFEKGD SQKIIVYIQA LGNTAHPRLL KTFEPYLEGK KSASRFQRLL MVASLYQMTR VHPTTARAVL YRIYKNPGEA |
| 111057493 | HLTSKHDQSL HQEYMPYLEE KLKSAFEKGD SQKIIVYIQA LGNTAHPRLL KTFEPYLEGK KSASRFQRLL MVASLYQMTR VHPTTARAVL YRIYKNPGEA |
| 111061289 | HLTSKHDQSL HQEYMPYLEE KLKSAFEKGD SQKIIVYIQA LGNTAHPRLL KTFEPYLEGK KSASRFQRLL MVASLYQMTR VHPTTARAVL YRIYKNPGEA |
| | 901 ... 1000 |
| 111061268 | AELRVAALHL LANANPSAAM LQRMAQQTHW EQSKEVISAT QSFIKSAARM DQNPNSIELA RNAQAAVDML NPNEYGSSLS KNFLSSFVID NIDKSYESQF |
| 111061279 | AELRVAALHL LANANPSAAM LQRMAQQTHW EQSKEVISAT QSFIKSAARM DQNPNSIELA RNAQAAVDML NPNEYGSSLS KNFLSSFVID NIDKSYESQL |
| 111057493 | AELRVAALHL LANANPSAAM LQRMAQQTHW EQSKEVISAT QSFIKSAARM DQNPNSIELA RNAQAAVDML NPNEYGSSLS KNFLSSFVID NIDKSYESQL |
| 111061289 | AELRVAALHL LANANPSAAM LQRMAQQTHW EPSKEVISAT QSFIKSAAGM DQNPNSIKLA RNAQAAVDML NPNEYGSSLS KNFLSSFVVD HIDKSYESQL |
| | 1001 ... 1100 |
| 111061268 | SSIGSVDSII PSSVFVNFMA NDGGYKHQVF HHSAMFSSVN DLLELVNTQF KNNNNNNSNN --RRNNKSGS HDNEDNHNNR NSNNEWTAEN VLKALNIQKD |
| 111061279 | SSIGSVDSII PSSVFVNFMA NDGGYKHQVF HHSAMFSSVN DLLELVNTQF KNNNNNN--- --RRNNKSGS HDNEDNHNNR NSNNEWTAEN VLKALNIQKD |
| 111057493 | SSIGSVDSII PSSVFVNFMA NDGGYKHQVF HHSAMFSSVN DLLELVNTQF KNNNNNN--- --RRNNKSGS HDNEDNHNNR NSNNEWTAEN VLKALNIQKD |
| 111061289 | SSIGSVDSII PSSVFVNFMA NDGGYKHQVF HHSAMFSSVN DLLELVNTQF KNNNNNNNNN NNRKNNKSGS YDNEDNHNNR NSNNEWTAEN VLKALNIQKN |
| | 1101 ... 1200 |
| 111061268 | QAEQLEGNVF LTMLGGKRAF AINNHTIEKI PSIFKEAAQK LKHTSFNLTQ FYSKNTMKVA FPTPMGLPFV YATSVPTMVY VGGETKVNSH PDLANGNSNF |
| 111061279 | QAEQLEGNFF LTMLGGKRAF AINNHTIEKI PSIFKEAAQK LKHTSFNLTQ FYSKNTMKVA FPTPMGLPFV YTSSVPTMVY VGGETKVNSH PDLANGNNNF |

111057493 QAEQLEGNFF LTMLGGKRAF SINNHTIEKI PSIFKEAAQK LKHTSFNLTQ FYSKNTMKVA FPTPMGLPFV YTSSVPTMVY VGGETKVNSH PDLANGNNNF

111061289 QAEQLEGNFF LTMLGGKRAF AINNHTIEKI PSIFKETAQK LKHTSFNLTQ FYSKNTMKVA FPTPMGLPFV YATSVPTMVY VGGETKVNSH PDLANGNNNF

1201 1300

111061268 VNIPQYINIT ADIEAVYSMQ FNSKFGMVAP FNHHEYYASV EKNMQCYLAV QTEANIDLEN NEVEFTVQPL NKEDKQNVFQ YSSVLYTTKS NILNFNPALQ

111061279 VNIPQYINIS ADIEAVYSMQ SNSKFGMVSP FNHHEYYASV EKNMQFYMAV QTEANIDLEN SEVEFTVQPL NKEDKQNVFQ YSTVLYTTKS NILNFNPALQ

111057493 VNIPQYINIS ADIEAVYSMQ SNSKFGMVTP FNHHEYYASV EKNMQFYMAV QTEANIDLEN NEVEFTVQPL NKEDKQNVFQ YSSVLYTTKS NILNFNPALQ

111061289 VNIPQYINIS ADIEAVYSMQ ANSKFGVVAP FNHHEYYASV EKNMQFYMAV QTEANIDLEN NEVEFTVQPL NKEDKQNVFQ YSTVLYTTKS NILNFNPALQ

1301 1400

111061268 EDGTERVHVG KAKQIQMNFG KESTGFAFEA NYWSENGFGD FASLYNEVSK FDFQSAMTSP WAQGSINSNN ITVAFNPRQS TSQVAKFTFS YADNSDDNNN

111061279 EDGTERVHVG KAKQIQMNFG KESTGFTFEA NYWSENGFGD FASLYNEVSK FDFQSAMTSP WAQGSINSNN ITVAFNPRQS TSQVAKFTFS YADNSDDNNN

111057493 EDGTERVHVG KAKQIQMNFG KESTGFAFEA NYWSENGFGD FASLYNEVSK FDFQSAMTSP WAQSSINSNN ITVAFNPRQS TSQVAKFTFS YADNSDDNNN

111061289 EDGTERVHVG KAEQIEMKFG KESTGFAFEA SYWSENGYGD FATLYNEVSK FDFQSAMTSP WAQGSLNSNN ITVAFNPRQS TSQVAKFTFS YAENSDDNNN

1401 1500

111061268 SHSGHDSNSS NNNNNNRADY SDAQPSSTAA NSRSRQNEFL RKAAAGISGA DAMVVDVSAR FQDSHGQSNA QYVATVAMAN SDASPNARML FFASMNPANS

111061279 SHSGHDSNSS NNNNNNRADY SDAQPSSTAA NSRSRQNEFL RKAAAGISGA DAMVVDVSAR FQDSHGQSNA QYVATVAMAN SDASPNARML FFASMNPANS

111057493 SHSGHDSNNS NNNNNNRADY TDAQPSSTAA NSRSRQNEFL RKAAAGISGA DAMVVDVSAR FQDSHGQSNA QYVATVAMAN SDASPNARML FFASMNPANS

111061289 SHSGHDSNSS NNNNNNRADY SDAQPSSTAA NSRSRQNEFL RKAAAGISGA DAVVVDVSAR FQGSHGQSNA QYVATVAMAN SDASSNARML FFASMNPANS

1501 1600

111061268 DSKAQVCAAA ASNFPNVPLM NFHDALKANP TSRISADIAF GAQCNAGGHI HADAKLSQTQ EFQEYTKSRP MAKKCFQLME KGQALEYACQ NATKVANMLN

111061279 DSKAQVCAAA ASNFPNVPLM NFHDALKANP TSRISADIAF GAQCNAGGHI HADAKLSQTQ EFQEYAKSRP MAKKCFQLME KGQALEYACQ NATKVANMLN

111057493 DSKAQVCAAA ASNFPNVPLM NFHDALKANP TSRISADIAF GAQCNAGGHI HADAKLSQTQ EFQEYAKSRP MAKKCFQLME KGQALEYACQ NATKVANMLN

111061289 DSKAQVCAAV ASNFPNVPLM NFLDALKANP TSHISADIAF GAQCNAGGHI HADARLSQTQ EFQEYAKSRP MAKKCFQLME KGQALEYACQ NATKVANMLN

1601 1700

111061268 NYEVSVKYDR VSSVFKNVTY SIYSALAQAA YPYHSENMFS QNSNPSGKID LNARFNYNLR YFNASINTPF FSANVKNVEV HHALRPLVIF HPSLNSLELM

111061279 NYEVSVKYDR VSSVFKNVTY SIYSALAQAA YPYHSENMFS QNSNPSGKID LNARFNYNLR YFNASINTPF FSANVKNVEV HHTLRPLVIF HPSLNSLELM

111057493 NYEVSVKYDR VSSVFKNVTY SIYSALAQAA YPYHSENMFS QNSNPSGKID LNARFNYNLR YFNASINTPF FSANVKNVEV HHALRPLVIF HPSLNSLELM

111061289 NYDVSVKYDR VPNALKNITY SIYSALAQAA YPYHNKNMFS QNSNPAGRIE ANVRFNNNLH YFNASINTPF FSGNVKNVEV DPALRPLVIF HPSLNSFELM

1701 1800

111061268 SYNENYDYPT CSVSKNSIST FDNKTYSADL EGWHVMFAST PKNYNDNSGR YSASNSQSNS FYKYKKVVVL AKNAGSQRKA VKMLLGENVI DINPSGSESS

111061279 SYNENYDYPT CSVSKNSIST FDNKTYSADL EGWHVMFAST PKNYNDNSGR YSASNSQSNS FYKYKKVVVL AKNAGSQRKA VKMLLGENVI DINPSGSESS

111057493 SYNENYDYPT CSVSKNSIST FDNKTYSADL EGWHVMFAST PKNYNDNSGR YSASNSQSNS FYKYKKVVVL AKNAGSQRKA VKMLLGENVI DINPSGSESS

111061289 SYNENYDYPT CSVSKNSIST FDNKTYSADL EGWHVMFAST PKNYNDNSGR YSASNSQSNS FYKYKKVVVL AKNAGSERKA VKMLLGENVI DINPSGSESS

1801 1900

111061268 DNSPNANVQV NGNKVQIANN RMASFDDFDG ETLVEISVTD NGEVQVQSSS HGIAVYHDGA NFIIDADSYH RGEVRGLCGT YSGDKYTDFT TPNKCIMREA

111061279 DNSPNANVQV NGNKVQIANN RMASFDDFDG ETLVEISVTD NGEVQVQSSS HGIAVYHDGA NFIIDADSYH RGEVRGLCGT YSGDKYTDFT TPNKCIMREA

111057493 DNSPNANVQV NGNKVQIANN RMASFDDFDG ETLVEISVTD NGEVQVQSSS HGIAVYHDGA NFIIDADSYH RGEVRGLCGT YSGDKYTDFT TPNKCIMREA

111061289 DNSPNANVQV NGNKVQITNN RMTSFDDFDG ETLVEISVTD NGEVQVQSSS HGIAVYHDGA NFIIDADSYH RGEVRGLCGT YSGDKYTDFT TPNKCIIREA

1901 2000

111061268 RLFAATYALP GSSNSNVEQL KRQADQMTCF RRRHIFANVI TSNDYDRSSS SSSSSNRNNN RNNNKNNRSN NNSSERLANP TKLIQDVKNN GDQVCISIRP

111061279 RLFAATYALP GSSNSNVEQL KRQADQMPCF RRRHIFANVI TSNDYDRSSS -------NSN ---------N NHSSERFINP TKLVQDIKNN LDRLCFSIHP

111057493 RLFAATYALP GSSNSNVEQL KRQADQMPCF RRRHIFANVI TSNDYDRSSS SS---NRNNN ---------K NHSSERLANP TKLVQDIKNN GDQVCFSIHP

111061289 RLFAATYALP GSSNSNVEQL KRQADQMPCL RRRHIFADVI TSNDYDRSSS SSSSSSNRNN ---NKNNRNN NNSSELRTNP TKLVQDIKNN GDHVCFSIHP

2001 2065

111061268 VPKCQKGFSP AGSSEKEVDY VCMSHGKNAQ FWINQIFQGG YVKLEQKQHN ATFMKNIPQR CVRDN

111061279 VPKCQSGFSP AESSEKEFQY FCMDQGKNAQ YWANQIRNGG FVNLEQKQPN ATFKKNIPKS CVRDN

111057493 VPKCQSGFSP AGSSEKEVQY FCMGQGKNSE YWVGQIINGG FVNLGQKQPN ATFKKSVPKS CVRDN

111061289 VPKCQSGFSP AESSEKEVQY LCISKGKNAE YWVGQISNGG FVNLGQKQPN ATFKKSVPKS CVRDN
