# Supplementary figures and images for: Role of nuclear protein Akirin in the modulation of female reproduction in Nilaparvata lugens (Hemiptera: Delphacidae)
Source: Front Physiol. 2024 Jul 9;15:1415746. doi: 10.3389/fphys.2024.1415746 (PMC11264338; doi:10.3389/fphys.2024.1415746)

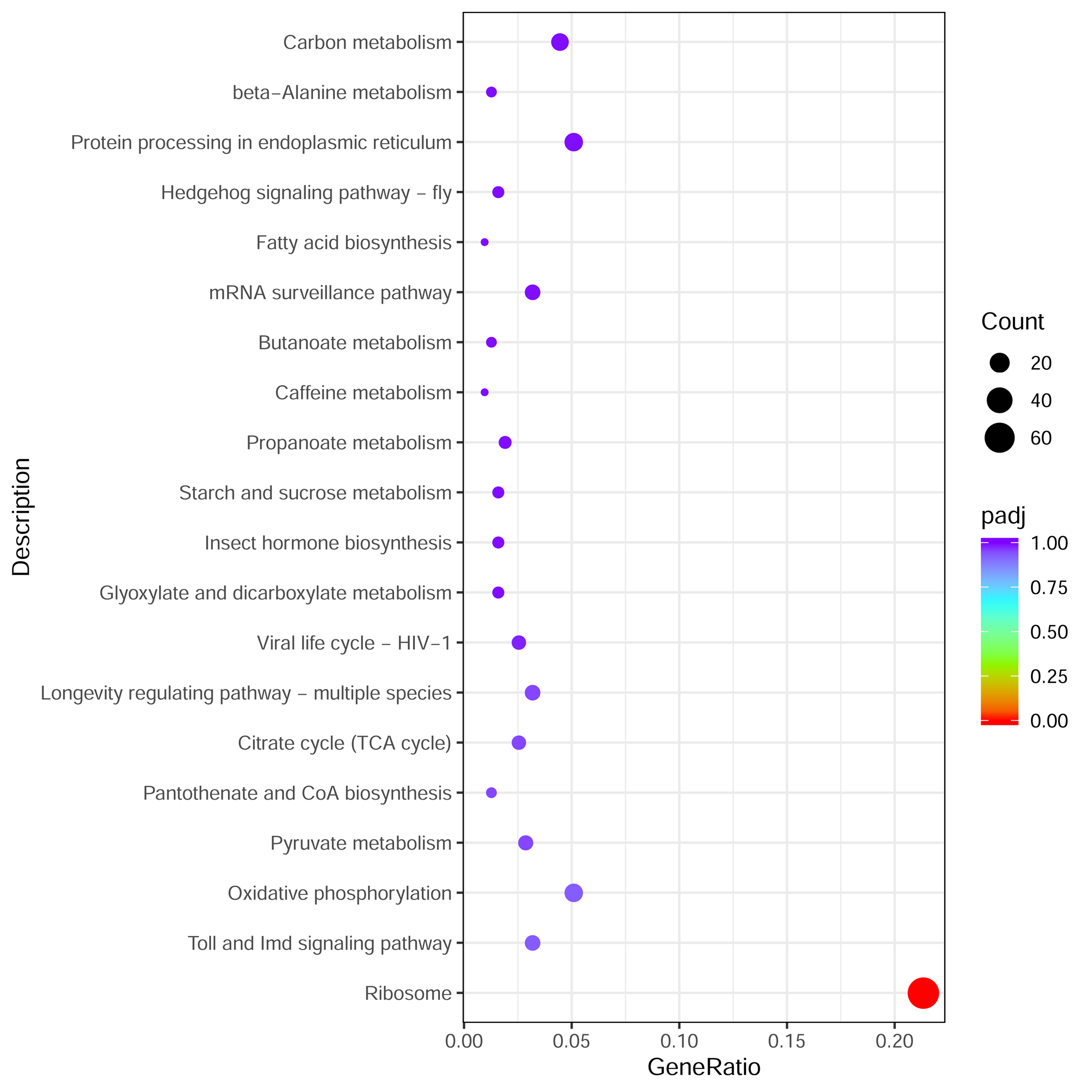

Supplement: Supplementary file 3 [file Image3.TIF]

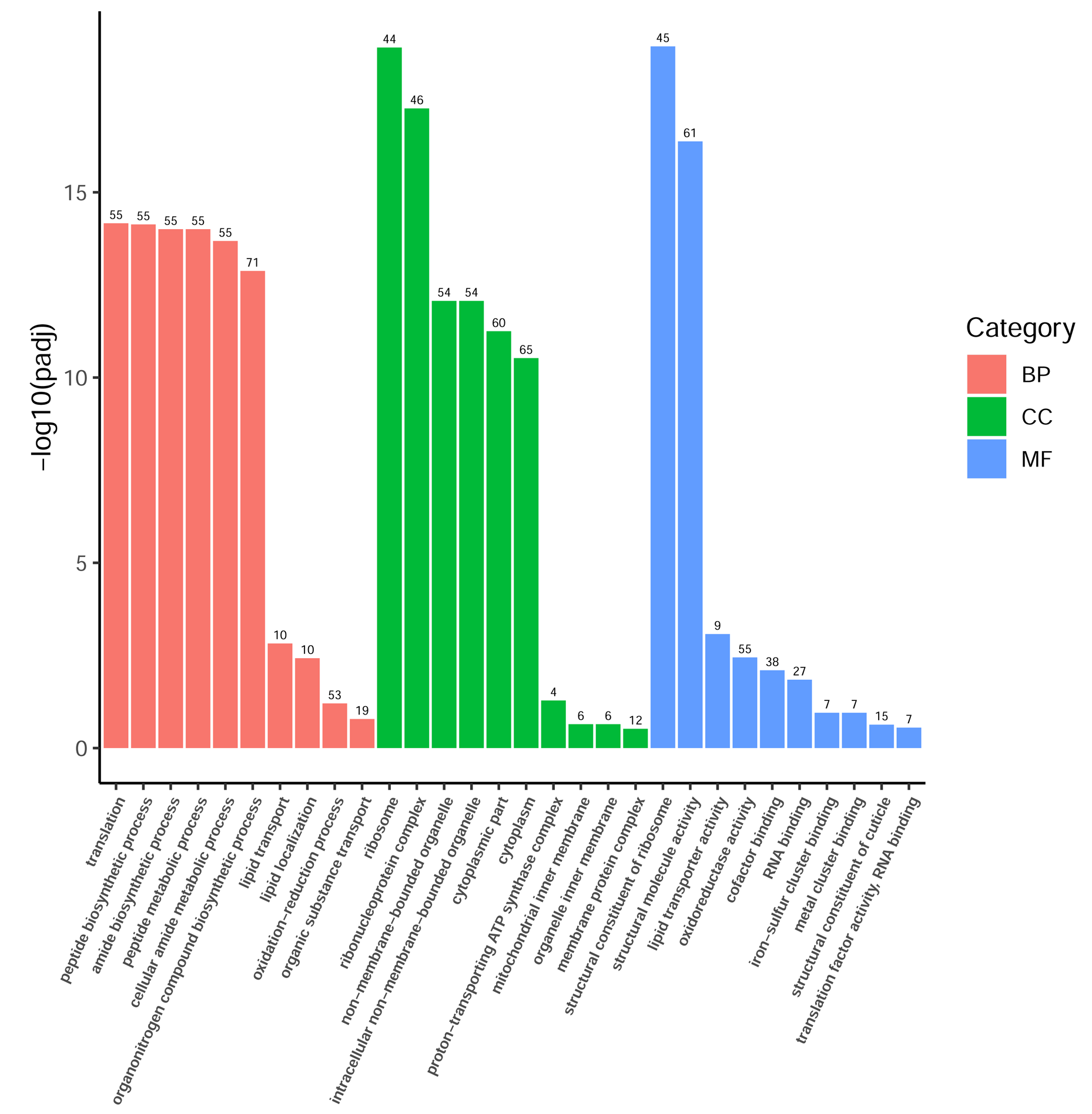

Supplement: Supplementary file 5 [file Image2.TIF]

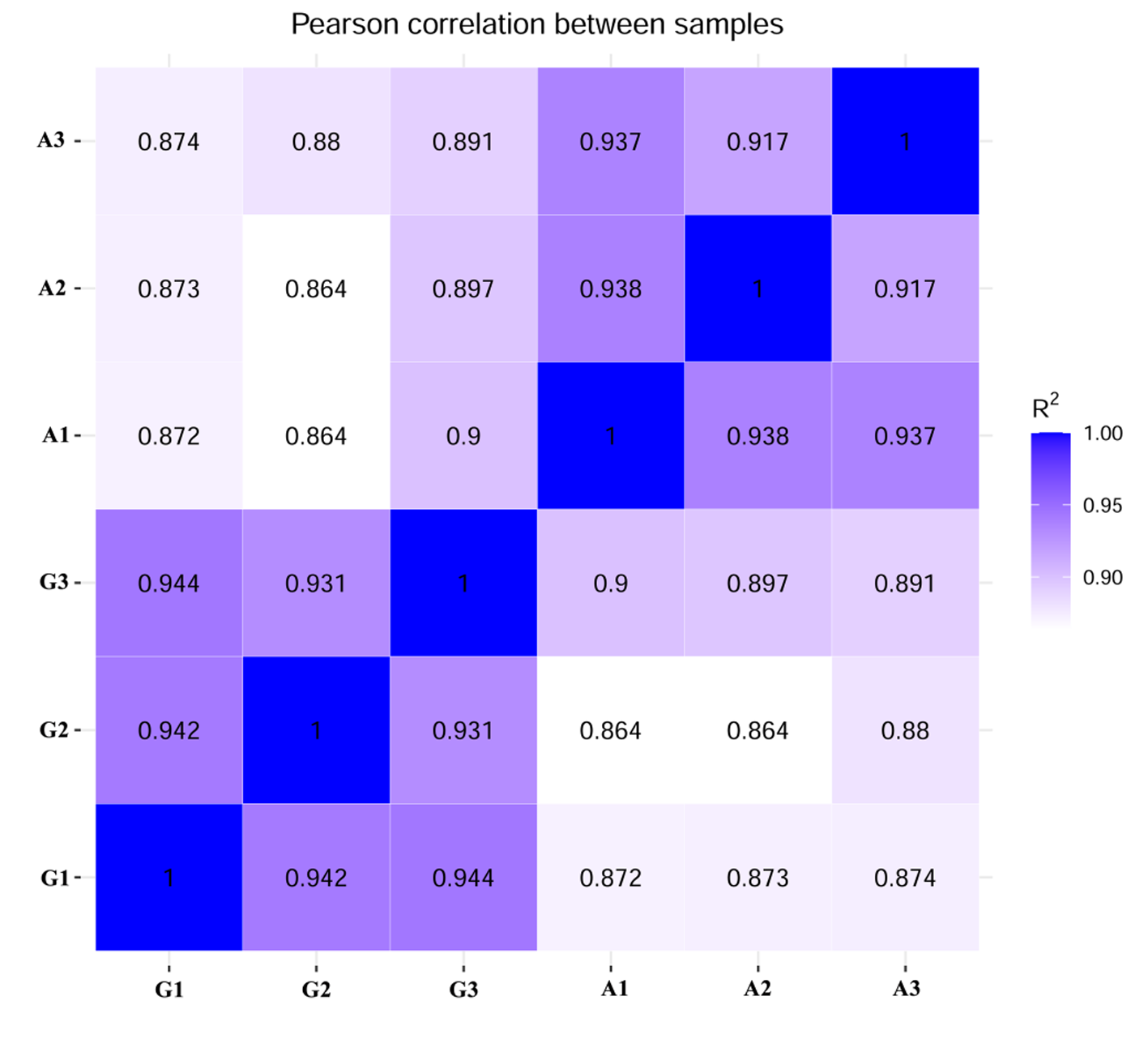

Supplement: Supplementary file 6 [file Image1.TIF]
