# Supplementary material for: Role of nuclear protein Akirin in the modulation of female reproduction in Nilaparvata lugens (Hemiptera: Delphacidae)
Source: Front Physiol. 2024 Jul 9;15:1415746. doi: 10.3389/fphys.2024.1415746 (PMC11264338; doi:10.3389/fphys.2024.1415746)
Supplement: Supplementary file 4 [file Table3.DOC]

| Sample | raw reads | clean reads | clean bases | error rate% | Q20% | Q30% | GC pct |
| --- | --- | --- | --- | --- | --- | --- | --- |
| ds*GFP1* | 43517382 | 41842588 | 6.28 G | 0.03 | 97.25 | 92.33 | 35.21 |
| ds*GFP2* | 43977042 | 42653324 | 6.4 G | 0.03 | 97.46 | 92.85 | 37.31 |
| ds*GFP3* | 43555832 | 41943930 | 6.29 G | 0.03 | 97.29 | 92.35 | 34.45 |
| ds*Akirin1* | 43700732 | 41244026 | 6.19 G | 0.03 | 97.36 | 92.5 | 35.29 |
| ds*Akirin2* | 43306340 | 41213314 | 6.18 G | 0.03 | 97.16 | 92.1 | 36.09 |
| ds*Akirin3* | 42820808 | 39306220 | 5.9 G | 0.03 | 97.39 | 92.6 | 36.19 |
