# Supplementary material for: Role of nuclear protein Akirin in the modulation of female reproduction in Nilaparvata lugens (Hemiptera: Delphacidae)
Source: Front Physiol. 2024 Jul 9;15:1415746. doi: 10.3389/fphys.2024.1415746 (PMC11264338; doi:10.3389/fphys.2024.1415746)
Supplement: Supplementary file 7 [file DataSheet1.PDF]

```
Gene_ID    1                                                                                                      100
111061268  ATGCAAGGAC TAACTCTGCT TGTCGCCGTT CTCGCGGTTT CCGGAGTTTC TGCCAGC--- ---GGACCAT GGAACAGCAA CCAGCAGTAC CGCTACCATG
111061279  ATGAACGGAC TAACTCTGCT TCTCTGCGCC ATCGCGGTTG CCGGAGTATC TGCCAGCGGC AGTGGACCAT GGAACAGCAA CCAGCAGTAC CGCTACCATG
111057493  ATGAACGGAC TAACTCTGCT TCTCTGCGCC ATCGCGGTTG CCGGAGTATC TGCCAGCAGC AGTGGACCAT GGAACAGCAA CCAGCAGTAC CGCTACCATG
111061289  ATGAAGGGAA TAACTCTGAT TTTCTGCGTT ATCGCAGTTG CCGGAGTATC TGCCAGCGGA AACGGACCAT GGAACAGTAA CCAGCAGTAC CGCTACCATG
           101                                                                                                    200
111061268  TGCAAGGCAG ATCACTGTCA GCCATGCACC AGTCAGGATC AAACCAGTAC GTGGGCATGC ACCTGAGAGC AGAATTGGAA GTTGAGGCGA AGAATGAGAA
111061279  TGCAAGGCAG ATCACTGTCA GCCATGCACC AGTCAGGATC AAACCAGTAC GTGGGCATGC ACCTGAGAGC AGAATTGGAA GTTGAGGCGA AGAATGAGAA
111057493  TGCAAGGCAG ATCACTGTCA GCCATGCACC AGTCAGGATC AAACCAGTAC GTGGGCATGC ACCTGAGAGC AGAATTGGAA GTTGAGGCGA AGAATGAGAA
111061289  TGCAAGGCAG ATCACTGTCA GCCATGCACC AGTCAGGATC AAACCAGTAC GTGGGCATGC ACCTGAGAGC AGAATTGGAA GTTGAGGCGA AGAATGAGAA
           201                                                                                                    300
111061268  CCAGGCTGTG TTCAAGATCT CCAAGGCCGA GTACGCCGAT GTGCACCAGA ACCTAAGTGG AGGCTGGCAA CAAGAGTTAC GCAGCAATGA GCTGCAGTAC
111061279  CCAGGCTGTG TTCAAGATCT CCAAGGCCGA GTATGCCGAT GTGCACCAGA ACCTGAGCGG AGGCTGGCAA CAAGAGTTAC GCAGCAATGA GCTGCAGTAC
111057493  CCAGGCTGTG TTCAAGATCT CCAAGGCCGA GTACGCCGAT GTGCACCAGA ACCTAAGCGG AGGCTGGCAA CAAGAGTTAC GCAGCAATGA GCTGCAGTAC
111061289  CCAGGCTGTG TTCAAGATCT CCAAGGCCGA GTACGCCGAT GTGCACCAGA ACCTGAGTGG AGGCTGGCAA CAAGAGTTAA GCAGCAATGA GCTGCAGTAC
           301                                                                                                    400
111061268  AAGCAGTTGC CCCTGTCGCA AGCAAACCAA GTCTTTCAAG TCAACTACAA GCAGGGAGCA GTGCGTAGTC TGCAGGTCAA CAGGAACACA CCCACCTGGG
111061279  AAGCAGTTGC CCCTGTCGCA AGCAAACCAA GTCTTTCAAG TCAACTACAA GCAGGGAGCA GTGCGCAGTC TGCAGGTCAA CAGGAACACA CCCACCTGGG
111057493  AAGCAGTTGC CCCTGTCGCA AGCAAACCAA GTCTTTCAAG TCAACTACAA GCAGGGAGCA GTGCGCAGTC TGCAGGTCAA CAGGAACACA CCCACCTGGG
111061289  AAGCAGTTGC CCCTGTCACA AGCAAACCAA GTCTTTCAAG TCAACTACAA GCAGGGAGCA GTGCGCAGTC TGCAGGTCAA CAGGAACACA CCCACCTGGG
           401                                                                                                    500
111061268  AACTGAACAT GATCAAGGGA TTCGTCAGCC TGTTCCAGGT CGATGTGATG GCCNAGAATG CCATCAAGTC TAGGCGCAAC ATTGTGCCCA ATGGCCAGCA
111061279  AACTGAACAT GATCAAGGGA TTCGTCAGCC TGTTCCAGGT CGATGTGACT GGCCAGAATG CCATCAAGTC TAGGCGCAAC ATTGTGCCCA ATGGCCAGCA
111057493  AACTGAACAT GATCAAGGGA TTCGTCAGCC TGTTCCAGGT CGATGTGACT GGCCAGAATG CCATCAAGTC TAGGCGCAAC ATTGTGCCCA ATGGCCAGCA
111061289  AACTGAACAT GATCAAGGGA TTTGTCAGCC TGTTCCAGGT CGATGTGACT GGCCAGAATG CCATCAAGTC TAGGCGCAAC ATTGTGCCCA ATGGCCAGCA
           501                                                                                                    600
111061268  GGTGAGTGGC TCGTTCAAGG TTATGGAGGA CTCTGTCACC GGCAAGTGTG AGACCCACTA CGATGTTGAT GAGCTGCCAA TGAGAGTTGT GCAACAGCAC
111061279  GGTGAGTGGC TCGTTCAAGG TTATGGAGGA CTCTGTCACC GGCAAGTGTG AGACCCACTA CGATGTTGAT GAGCTGCCAA TGAGAGTTGT GCAACAGCAC
111057493  GGTGAGTGGC TCGTTCAAGG TTATGGAGGA CTCTGTCACC GGCAAGTGTG AGACCCACTA CGATGTTGAT GAGCTGCCAA TGAGAGTTGT GCAACAGCAC
111061289  GGTGAGTGGC TCGTTCAAGG TTATGGAGGA CTCTGTCACC GGCAAGTGTG AGACCCACTA CGATGTTGAT GAGCTGCCAA TGAGAGTTGT GCAACAGCAC
           601                                                                                                    700
111061268  CCAGAGATTG CACCACTTGC AGTTAAGCAG CAGGGCCAGG GCCAGGGCCA GGGCCAGAGC CACAGCCGTC TCATTCAGGT GGTCAAGTCA AGGAACTTTA
111061279  CCAGAGATTG CACCACTTGC AGTTAAGCAG CAGGGCCAGG GCCAGGGCCA G------AGC CACAGCCGTC TCATTCAGGT GGTCAAGTCA AGGAACTTTA
111057493  CCAGAGATTG CACCACTTGC AGTTAAGCAG CAGGGCCAGG GCCAGGGCCA G------AGC CACAGCCGTC TCATTCAGGT GGTCAAGTCA AGGAACTTTA
111061289  CCAGAGATTG CACCACTTGC AGTTAAGCAG CAGGGCCAGG GCCAGGGCCA G------AGC CACAGCCGTC TCATTCAGGT GGTCAAGTCA AGGAACTTTA
           701                                                                                                    800
111061268  GCAACTGTGA TAACCCAGTC ACCTACCACT TTGGATTCAC TCAGGAAACC AACTTTGAGC CAGCCAGCAA CCAGATGGGC AACCTTGTCA GCCGTGCCGC
111061279  GCAACTGTGA TAACCCAGTC ACCTACCACT TTGGATTCAC TCAGGAAACC AACTTTGAGC CAGCCAGCAA CCAGATGGGC AACCTTGTCA GCCGTGCCGC
111057493  GCAACTGTGA TAACCCAGTC ACCTACCACT TTGGATTCAC TCAGGAAAGC AACTTTGAGC CAGCCAGCAA CCAGATGGGC AACCTTGTCA GCCGTGCCGC
111061289  GCAACTGTGA TAACCCAGTC ACCTACCACT TTGGATTCAC TCAGGAAAGC AACTTTGAGC CAGCCAGCAA CCAGATGGGC AACCTTGTCA GCCGTGCCAC
           801                                                                                                    900
111061268  AATGGGCCAT ATGATTATTG CTGGAGAAAT AGAAAGTTTC ACCATCCACT CATCGGTCAC TCAGAATGAG ATTGCCATCA GTCCATTCGG CTACAACCAG
111061279  AATGGGCCAT ATGATTATTG CTGGAGAAAT AGACAGTTTC ACCATCCACT CATCGGTCAC TCAGAATGAG ATTGCCATCA GTCCATTCGG CTACAACCAG
111057493  AATGGGCCAT ATGATTATTG CTGGAGAAAT AGAAAGTTTC ACCATCCACT CATCGGTCAC TCAGAATGAG ATTGCCATCA GTCCATTCGG CTACAACCAG
111061289  AACGAGCCGT ATCCTTCTTG CTGGAGAACC AGACAGTTAC ACCATCCACT CATCAGTCAC TCAGAATGAG ATTGCCATCA GTCCATTCGG CTACAACCAG
           901                                                                                                   1000
111061268  CAGAAGGGAG TAGTTGGCAC ACTCATGAAT GCCACCCTTG TCTCCGTGTC ACATGCCTCT TCTGGCTCTC CTCAGTCAGT CCAGAACCCA CAGAAAATCA
111061279  CAGAAGGGAG TAGTTGGCAC ACTCATGAAT GCCACCCTTG TCTCCGTGTC ACATGCCTCT TCTGGCTCTC CTCAGTCAGT CCAGAACCCA CAGAAAATCA
111057493  CAGAAGGGAG TAGTTGGCAC ACTCATGAAT GCCACCCTTG TCTCCGTGTC ACATGCCTCT TCTGGCTCTC CTCAGTCAGT CCAGAACCCA CAGAAAATCA
111061289  CAGAAGGGAG TAGTTGGCAC ACTCATGAAT GCCACCCTTG TCTCCGTGTC ACATGCCTCT TCTGGCTCTC CTCAGTCAGT CCAGAACCCA CAGAAAATCA
           1001                                                                                                  1100
111061268  ACGATCTGGT TTATGAGTTC AACCCAGCAT CAAACAGTGA GAGCAACCAA AGATCTAGCC ACTACACCAG GCAGCAGGCT GATAATGAAG ATGACAGCAG
111061279  ACGATCTGGT TTATGAGTTC AACCCAGCAT CAAACAGTGA GAGCAACCAA AGATCTAGCC ACTACACCAG GCAGCAGGCT GATAATGAAG ATGACAGCAG
111057493  ACGATCTGGT TTATGAGTTC AACCCAGCAT CAAACAGTGA GAGCAACCAA AGATCTAGCC ACTACACCAG GCAGCAGGCT GATAATGAAG ATGACAGCAG
111061289  ACGATCTGGT TTATGAGTTC AACCCAGCAT CAAACAGTGA GAGCAACCAA AGATCTAGCC ACTACACCAG GCAGCAGGCT GATAATGAAG ATGACAGCAG
           1101                                                                                                  1200
111061268  CTCAAGCTCA TCTAGCAGTG ACTCTTCTTC ATCATCCTCC TCCTCTTCAT CTAGCTCATC ATCATCATCG AGCAGCTCAG AGGAGAACAA CAAGAATAGT
111061279  CTCAAGCTCA TCTAGCAGTG ACTCTTCTTC ATCATCCTCC TCCTCTTCAT CTAGCTCATC ATCATCATCG AGCAGCTCAG AGGAGAACAA CAAGAATAGT
```

```
111057493  CTCAAGCTCA TCTAGCAGTG ACTCTTCTTC ATCATCCTCC TCCTCTTCAT CTAGCTCATC ATCATCATCG AGCAGCTCAG AGGAGAACAA CAAGAATAGT
111061289  CTCAAGCTCA TCTAGCAGTG ACTCTTCTTC ATCATCCTCC TCCTCTTCAT CTAGCTCATC ATCATCATCG AGCAGCTCAG AGGAGAACAA CAAGAGTAGT
           1201                                                                                                    1300
111061268  AAGAAGAACA ACAA------ ---------C AACAAGAACT GGAACAACAA GAACCAGAAG AAGAACAACA ACAACAACAG GAACAACCAT AATGACAATG
111061279  AAGAAGAACA ACAAGAACTG GAACAACAAC AACAAGAACT GGAACAACAA GAACCAGAAG AACAACAACA ACAACAACAG GAACAACCAT AATGACAGTG
111057493  AAGAAGAACA ACAA------ ---------C AACAAGAACT GGAACAACAA GAACCAGAAG AAGAAGAACA ACAACAACAG GAACAACCAT AATGACAATG
111061289  AAGAAGAACA ACAT------ ---------C AACAAGAACT GGAACAACAA GAACCAGAAT AAGAACAACA ACAATAACAG GAACAACCAT AATAACAATG
           1301                                                                                                    1400
111061268  ACAACAACCA GGATAACTCC AATGAAAACA ATAATGATGA TGCTTACTGG AGGAGCCAGC AGAAGACCAA GTCCAGGTCA CGCAGGAGTA TTCTGAGGAA
111061279  ACAACAACCG GGATAACTCC AATGAAAACA ATAATGATGA TGCTTACTGG AGGAGCCAGC AGAAGACCAA GTCCAGGTCA CGCAGGAGTA TTCTGAGGAA
111057493  ACAACAACCA GGATAACTCC AATGAAAACA ATAATGATGA TGCTTACTGG AGGAGCCAGC AGAAGACCAA GTCCAGGTCA CGCAGGAGTA TTCTGAGGAA
111061289  ACAACAACCA GGATAACTCC AATGAAAACA ATAATGATGA TGCTTACTGG AGGAGCCAGC AGAAGACCAA GTCCAGGTCA CGCAGGAGTA TTCTGAGGAA
           1401                                                                                                    1500
111061268  CTACAACAAT GAGAATGATG ATGACAACAA CCAAAACAGG AACCAGAACA GAAACAACAA CAACAACAAC AA------TG ACTCCTCTGA AGAGAGCAAT
111061279  CTACAACAAC GAGAATGATT ATGACAACAA CCAAAACAGG AACCAGAACA GAAACAACAA CAACAACAAC AACAACAATG ACTCCTCTGA AGAGAGCAAT
111057493  CTACAACAAC GAGAATGATG ATGACAACAA CCAAAACAGG AACCAGAACA GAAACAACAA CAACAACAAC AACAA---TG ACTCCTCTGA AGAGAGCAAT
111061289  CTACAACAAC GAGAATGATG ATGACCACAA CCAAAACAGA AACCAGAACA GAAACAACAA CAACAACAAC AA------TG ACTCCTCTGA AGAGAGCAAT
           1501                                                                                                    1600
111061268  GAAAACCAGA ACAACAA--- ------CAAG AACTGGAACA ACAATAACAA TAACAATAAC AAGAACTGGA ACAACAATGA CAACAA---G AACTGGAACA
111061279  GAAAACCAGA ACAACAA--- ---------- --CAAGAACT GGAACAACAA TAACAATAAC AAGAACTGGA ACAACAATGA CAACAA---G AACTGGAACA
111057493  GAAAACCAGA ACAACAATAA CAAGCACAAC AACAAGAACT GGAACAACAA TGACAACAAC AAGAACTGGA ACAACAATGA CAACAA---- --CAAGAACT
111061289  GAAAACCAGA ACAACAATAA CAAGCACAAC AACAAGAACT GGAACAACAA TGACAACAAC AAGAACTGGA ACAACAATGA CAACAACAAG AACTGGAACA
           1601                                                                                                    1700
111061268  ACAATGACAA CAACAA---- ---------- -------CAA GAACAGGAAC AACAATGACA ATGATGACTC ATCATCAAGC TCAAGCAGTT CCAGTTCAAG
111061279  ACAATGACAA CAACAA---- ---------- -------CAA GAACAGGAAC AACAATAACA ATGATGACTC ATCATCAAGC TCAAGCAGTT CCAGTTCAAG
111057493  GGAA---CAA CAACAA---- ---------- -------CAA GAACAGGAAC AACAATGACA ATGATGACTC ATCATCAAGC TCAAGCAGTT CCAGTTCTAG
111061289  ACAATGACAA CAACAAGAAC TGGAACAACA ACAACAACAA GAACAGGAAC AACAATGACA ATGATGACTC ATCATCAAGC TCAAGCAGTT CCAGTTCTAG
           1701                                                                                                    1800
111061268  CTCCTCCTCC TCGTCCTCAT CTTCATCCTC TTCGTCCTCA TCCTCTGATC TTGACAGCAG CGAAGAGAAC TGGCAGCAGA AGCCTGGCAT GAATGATGCA
111061279  CTCCTCCTCC TCGTCATCAT CTTCATCCTC TTCGTCCTCA TCCTCTGATC TTGACAGCAG CGAAGAGAAC TGGCAGCAGA AGCCTGGCAT GAATGATGCA
111057493  CTCCTCCTC- --GTCCTCAT CTTCATCCTC TTCGTCCTCA TCCTCTGATC TTGACAGCAG CGAAGAGAAC TGGCAGCAGA AGCCTGGCAT GAATGATGCA
111061289  CTCCTCCTCC TCGTCCTCAT CTTCATCCTC TTCGTCCTCA TCCTCTGATC TTGACAGCAG CGAAGAGAAC TGGCAGCAGA AGCCTGGCAT GAATGATGCA
           1801                                                                                                    1900
111061268  CCAAGAACAC CTTTCTTGCC CCACTTTGTT GGAGTAAGAG GCAACTCAAT TCAGGCTGAC AAGCAGGTCG ACATTGTCAA TGAAGTCCAA AAGGTAGCCA
111061279  CCAAGAACAC CTTTCTTGCC CCACTTTGTT GGAGTAAGAG GCAACTCAAT TCAGGCTGAC AAGCAGGTCG ACATTGTCAA TGAAGTCCAA AAGGTAGCCA
111057493  CCAAGAACAC CTTTCTTGCC CCACTTTGTT GGAGTAAGAG GCAACTCAAT TCAGGCTGAC AAGCAGGTCG ACATTGTCAA TGAAGTCCAA AAGGTAGCCA
111061289  CCAAGAACAC CTTTCTTGCC CCACTTTGTT GGAGTAAGAG GCAACTCAAT TCAGGCTGAC AAGCAGGTCG ACATTGTCAA TGAAGTCCAA AAGGTAGCCA
           1901                                                                                                    2000
111061268  TGAGGATTGG CGCCCAGGTC CAGAGACCCA GTGCCATTCC AGGACAGAAC ACACTCACCT CCTTCACCAT TCTCACCAGG ATGATTCAGA CCATGTCAGC
111061279  TGAGGATTGG CGCCCAGGTC CAGAGACCCA GTGCCATTCC AGGACAGAAC ACACTTACCT CCTTCTCCAT TCTCACCAGG ATGATCCAGA CCTTGTCAGC
111057493  TGAGGATTGG CGCCCAGGTC CAGAGACCCA GTGCCATTCC AGGACAGAAC ACACTCACCT CCTTCACCAT CCTCACCAGG ATGATCCAGA CCATGTCAGC
111061289  TGAGGATTGG CGCCCAGGTC CAGAGACCCA GTGCCATTCC AGGACAGAAC ACACTCACCT CCTTCACCAT CCTCACCAGG ATGATCCAGA CCATGTCAGC
           2001                                                                                                    2100
111061268  CAAGCAGATC CAGGAAGTCA AGCAGAGACT TTTCATTGAC AGGAACAATG CCAATGGCAA GAGTTCTGCT GATGCCAAGA AACTCCAGAG TTGGGAAGCA
111061279  CAAGCAGATC CAGGAAGTCA AACAGAGACT TTTTATTGAC AGGAACAATG CCAATGGCAA GAGTTCTGCT GATGCCAAGA AACTCCAGAG TTGGGAAGCA
111057493  CAAGCAGATC CAGGAAGTCA AACAGAGACT TTTCATTGAC AGGAACAATG CCAATGGCAA GAGTTCTGCT GATGCCAAGA AACTCCAGAG TTGGGAAGCA
111061289  CAAGCAGATC CAGGAAGTCA AACAGAGACT TTTCATTGAC AGGAACAATG CCAATGGCAA GAGCTCTGCT GATGCCAAGA AACTCCAGAG TTGGGAAGCA
           2101                                                                                                    2200
111061268  TTCAAGCATG CTACAGCTAA TGCTGGAACT GGACCAGCAT TGGAAGCCAT CAAGAACTGG GTTGAGAAGG GTGATGTGAG GAATGAGAAG GCAGCTGAAC
111061279  TTCAAGCATG CTACAGCTAA TGCTGGAACT GGACCAGCAT TGGAAGCCAT CAAGAACTGG GTTGAGAAGG GTGATGTGAG GAATGAGAAG GCAGCTGAAC
111057493  TTCAAGCATG CTACAGCTAA TGCTGGAACT GGACCAGCAT TGGAAGCCAT CAAGAACTGG GTTGAGAAGG GTGATGTGAG GAATGAGAAG GCAGCTGAAC
111061289  TTCAAGCATG CTACAGCTAA TGCTGGAACT GGACCAGCAT TGGAAGCCAT CAAGAACTGG GTTGAGAAGG GTGATGTGAG GAATGAGAAG GCAGCTGAAC
           2201                                                                                                    2300
111061268  TTGTTGCTGT TCTGCCAAGA ACCGCCAGAC TGCCAACTGA CCAGTACATC AAGACTTTCT TCCAATTTGC CACCTCATCA AATGTGCAGA ACCAGAAGTA
111061279  TTGTTGCTGT TCTGCCAAGA ACCGCCAGAC TGCCAACTGA TCAGTACATC AAGACTTTCT TCCAATTTGC CACCTCATCA AATGTGCAGA ACCAGAAGTA
111057493  TTGTTGCTGT TCTGCCAAGA ACCGCCAGAC TGCCAACTGA CCAGTACATC AAGACTTTCT TCCAATTTGC CACCTCATCA AATGTGCAGA ACCAGAAGTA
111061289  TTGTTGCTGT TCTGCCAAGA ACCGCCAGAC TGCCAACTGA CCAGTACATC AAGACTTTCT TCCAATTTGC CACCTCATCA AATGTGCAGA ACCAGAAGTA
           2301                                                                                                    2400
```

111061268 CCTCAACTCC ACCATCATCC TTGGATTCTC TGAAATCCTG AGGAAAGCTC AAGTTGACTC AGACACCAAA CACATGCGCT TTGGAGTTCA CAGCTTTGGT
111061279 CCTCAACTCC ACCATCATCC TTGGATTCTC TGAAATCCTG AGGAAAGCTC AAGTTGACTC AGACACCAAA CACATGCGCT TTGGAGTTCA CAGCTTTGGT
111057493 CCTCAACTCC ACCATCATCC TTGGATTCTC TGAAATCCTG AGGAAAGCTC AAGTTGACTC AGACACCAAA CACATGCGCT TTGGAGTTCA CAGCTTTGGT
111061289 CCTCAACTCC ACCATCATCC TTGGATTCTC TGAAATCCTG AGGAAAGCTC AAGTTGACTC AGACACCAAA CACATGCGCT TTGGAGTTCA CAGCTTTGGT

2401 2500
111061268 CACTTGACAT CCAAGCATGA TCAATCCCTG CATCAGGAAT ACATGCCCTA CTTGGAAGAG AAACTCAAGA GTGCCTTTGA AAAGGGTGAT AGCCAGAAAA
111061279 CACTTGACAT CCAAGCATGA TCAATCCCTC NATCAGGAAT ACATGCCCTA CTTGGAAGAG AAACTCAAGA GTGCCTTTGA AAAGGGTGAT AGCCAGAAAA
111057493 CACTTGACAT CCAAGCATGA TCAATCCCTG CATCAGGAAT ACATGCCCTA CTTGGAAGAG AAACTCAAGA GTGCCTTTGA AAAGGGTGAT AGCCAGAAAA
111061289 CACTTGACAT CCAAGCATGA TCAATCCCTG CATCAGGAAT ACATGCCCTA CTTGGAAGAG AAACTCAAGA GTGCCTTTGA AAAGGGTGAT AGCCAGAAAA

2501 2600
111061268 TCATTGTCTA CATCCAAGCT CTTGGAAACA CTGCACACCC AAGACTATTG AAGACCTTTG AGCCCTACCT GGAAGGAAAG AAATCAGCAT CACGCTTCCA
111061279 TCATTGTCTA CATCCAAGCT CTTGGAAACA CTGCACACCC AAGACTATTG AAGACCTTTG AGCCCTACCT GGAAGGAAAG AAATCAGCAT CACGCTTCCA
111057493 TCATTGTCTA CATCCAAGCT CTTGGAAACA CTGCACACCC AAGACTATTG AAGACCTTTG AGCCCTACCT GGAAGGAAAG AAATCAGCAT CACGCTTCCA
111061289 TCATTGTCTA CATCCAAGCT CTTGGAAACA CTGCACACCC AAGACTATTG AAGACCTTTG AGCCCTACCT GGAAGGAAAG AAATCAGCAT CACGCTTCCA

2601 2700
111061268 GCGCCTCTTG ATGGTTGCCA GCCTCTACCA GATGACCCGT GTCCACCCAA CCACTGCCCG TGCTGTGCTC TACAGAATCT ACAAGAACCC AGGAGAAGCC
111061279 GCGCCTCTTG ATGGTTGCCA GCCTCTACCA GATGACCCGT GTCCACCCAA CCACTGCCCG TGCTGTGCTC TACAGAATCT ACAAGAACCC AGGAGAAGCC
111057493 GCGCCTCTTG ATGGTTGCCA GCCTCTACCA GATGACCCGT GTCCACCCAA CCACTGCCCG TGCTGTGCTC TACAGAATCT ACAAGAACCC AGGAGAAGCC
111061289 GCGCCTCTTG ATGGTTGCCA GCCTCTACCA GATGACCCGT GTCCACCCAA CCACTGCCCG TGCTGTGCTC TACAGAATCT ACAAGAACCC AGGAGAAGCC

2701 2800
111061268 GCCGAACTCA GAGTGGCTGC CCTACACCTT TTGGCCAATG CTAACCCATC TGCAGCCATG CTACAGAGGA TGGCCCAGCA GACCCACTGG GAGCAAAGCA
111061279 GCCGAACTCA GAGTGGCTGC CCTACACCTT TTGGCCAATG CTAACCCATC TGCAGCCATG CTACAGAGGA TGGCCCAGCA GACCCACTGG GAGCAAAGCA
111057493 GCCGAACTCA GAGTGGCTGC CCTACACCTT TTGGCCAATG CTAACCCATC TGCAGCCATG CTACAGAGGA TGGCCCAGCA GACCCACTGG GAGCAAAGCA
111061289 GCCGAACTCA GAGTGGCTGC CCTACACCTT TTGGCCAATG CTAACCCATC TGCAGCCATG CTACAGAGGA TGGCCCAGCA GACCCACTGG GAGCCAAGCA

2801 2900
111061268 AGGAAGTCAT CTCAGCTACT CAATCCTTCA TCAAGAGTGC TGCTCGTATG GATCAGAATC CCAACTCCAT TGAATTGGCA AGGAATGCTC AGGCAGCTGT
111061279 AGGAAGTCAT CTCAGCTACT CAATCCTTCA TCAAGAGTGC TGCTCGTATG GATCAGAATC CCAACTCCAT TGAATTGGCA AGGAATGCTC AGGCAGCTGT
111057493 AGGAAGTCAT CTCAGCTACT CAATCCTTCA TCAAGAGTGC TGCTCGTATG GATCAGAATC CCAACTCCAT TGAATTGGCA AGGAATGCTC AGGCAGCTGT
111061289 AGGAAGTCAT CTCTGCTACT CAATCCTTCA TCAAGAGTGC TGCTGGTATG GATCAGAATC CCAACTCCAT TAAATTGGCA AGGAATGCTC AGGCAGCTGT

2901 3000
111061268 TGACATGCTC AACCCCAATG AGTATGGAAG CTCCCTGTCC AAGAACTTCC TCAGCTCATT TGTCATTGAC AACATCGACA AGAGCTATGA AAGCCAATTC
111061279 TGACATGCTC AACCCCAATG AGTATGGAAG CTCCCTGTCC AAGAACTTCC TCAGCTCATT TGTCATTGAC AACATCGACA AGAGCTACGA AAGCCAATTA
111057493 TGACATGCTC AACCCCAATG AGTATGGAAG CTCCCTGTCC AAGAACTTCC TCAGCTCATT TGTCATTGAC AACATCGACA AGAGCTACGA AAGCCAATTA
111061289 TGACATGCTC AACCCTAATG AGTATGGAAG CTCCCTGTCC AAGAATTTCC TCAGCTCATT TGTTGTTGAC CACATTGACA AGAGCTACGA AAGCCAATTA

3001 3100
111061268 AGTTCCATTG GCAGTGTTGA CAGTATTATT CCGAGCAGTG TGTTTGTCAA CTTCATGGCC AATGATGGAG GATACAAGCA CCAAGTGTTC CATCACTCTG
111061279 AGCTCCATTG GCAGTGTTGA CAGTATTATT CCAAGCAGTG TGTTTGTCAA CTTCATGGCC AATGATGGAG GATACAAGCA CCAAGTGTTC CATCACTCTG
111057493 AGCTCCATTG GCAGTGTTGA CAGTATTATT CCAAGCAGTG TGTTTGTCAA CTTCATGGCC AATGATGGAG GATACAAGCA CCAAGTGTTC CATCACTCTG
111061289 AGCTCCATTG GCAGTGTTGA CAGTATTATT CCAAGCAGTG TGTTTGTCAA CTTTATGGCC AATGATGGAG GATACAAGCA CCAAGTGTTC CATCACTCTG

3101 3200
111061268 CTATGTTCTC CAGTGTCAAT GACCTTTTGG AGCTTGTGAA CACCCAATTC AAGAACAACA ACAACAACAA CAGCAACAAC ------CGCA GAAACAACAA
111061279 CTATGTTCTC CAGTGTTAAT GACCTTTTGG AGCTTGTGAA CACTCAATTC AAGAACAACA ACAACAACAA C--------- ------CGCA GAAACAACAA
111057493 CTATGTTCTC CAGTGTTAAT GACCTTTTGG AGCTTGTGAA CACTCAATTC AAGAACAACA ACAACAACAA C--------- ------CGCA GAAACAACAA
111061289 CTATGTTCTC CAGTGTCAAT GACCTTTTGG AGCTTGTGAA CACCCAATTC AAGAACAACA ACAACAACAA CAACAACAAC AACAACCGCA AAAACAACAA

3201 3300
111061268 GTCTGGATCT CATGACAATG AAGACAATCA TAACAACAGG AACTCAAACA ACGAATGGAC AGCTGAGAAT GTTCTCAAGG CCCTCAACAT CCAGAAGGAC
111061279 GTCTGGATCT CATGACAATG AAGACAACCA TAACAACAGG AACTCAAACA ACGAATGGAC AGCTGAGAAT GTTCTCAAGG CCCTCAACAT CCAGAAGGAC
111057493 GTCTGGATCT CATGACAATG AAGACAACCA TAACAACAGG AACTCAAACA ACGAATGGAC AGCTGAGAAT GTTCTCAAGG CCCTCAACAT CCAGAAGGAC
111061289 GTCTGGATCT TATGACAATG AAGACAACCA TAACAACAGG AACTCAAACA ACGAATGGAC AGCTGAGAAT GTTCTCAAGG CCCTCAACAT CCAGAAGAAC

3301 3400
111061268 CAGGCTGAAC AGCTGGAAGG GAACGTCTTC CTCACCATGC TGGGAGGAAA GCGTGCTTTT GCCATCAACA ACCACACCAT TGAGAAGATC CCATCCATTT
111061279 CAGGCTGAAC AGCTGGAAGG AAACTTCTTC CTCACCATGC TGGGAGGAAA GCGTGCTTTT GCCATCAACA ACCACACCAT TGAGAAGATC CCATCCATTT
111057493 CAGGCTGAAC AGCTGGAAGG AAACTTCTTC CTCACCATGC TGGGAGGAAA GCGTGCTTTT TCCATCAACA ACCACACCAT TGAGAAGATC CCATCCATTT
111061289 CAGGCTGAAC AGCTGGAAGG AAACTTCTTC CTCACCATGC TGGGAGGAAA GCGTGCTTTC GCCATCAACA ACCACACCAT TGAGAAGATC CCATCCATTT

3401 3500
111061268 TCAAGGAAGC AGCACAGAAA CTGAAGCACA CCTCATTCAA CCTGACTCAG TTCTACAGCA AGAACACAAT GAAAGTAGCC TTCCCCACCC CAATGGGTCT
111061279 TCAAGGAAGC AGCACAGAAA CTGAAGCACA CCTCATTCAA CCTGACTCAG TTCTACAGTA AGAACACAAT GAAAGTAGCC TTCCCTACCC CAATGGGTCT
111057493 TCAAGGAAGC AGCACAGAAA CTGAAGCACA CCTCATTCAA CCTGACTCAA TTCTACAGTA AGAACACAAT GAAAGTAGCC TTCCCTACCC CAATGGGTCT

111061289 TCAAGGAAAC AGCACAGAAA CTGAAGCACA CCTCATTCAA CCTGACTCAG TTCTACAGCA AGAACACAAT GAAAGTAGCC TTCCCCACCC CAATGGGTCT

3501 3600

111061268 GCCTTTTGTC TATGCCACCA GTGTACCCAC CATGGTGTAT GTAGGTGGAG AGACCAAGGT CAACTCTCAC CCAGACTTGG CCAATGGCAA CAGCAACTTC

111061279 GCCTTTTGTC TATACCTCCA GTGTACCCAC CATGGTGTAT GTAGGTGGAG AGACCAAGGT CAACTCTCAC CCAGACTTGG CCAATGGCAA CAACAACTTC

111057493 GCCTTTTGTC TATACCTCCA GTGTACCCAC CATGGTGTAT GTAGGTGGAG AGACCAAGGT CAACTCTCAC CCAGACTTGG CCAATGGCAA CAACAACTTC

111061289 ACCTTTTGTC TATGCCACCA GTGTACCCAC CATGGTGTAT GTAGGTGGAG AGACCAAGGT CAACTCTCAT CCAGACTTGG CCAATGGCAA CAACAACTTT

3601 3700

111061268 GTCAACATTC CTCAATACAT CAACATCACA GCTGACATCG AAGCAGTCTA CTCAATGCAG TTCAACAGCA AATTTGGTAT GGTTGCTCCA TTCAACCACC

111061279 GTCAACATTC CTCAATATAT CAACATCTCA GCTGACATTG AAGCAGTCTA CTCAATGCAG TCCAACAGCA AATTTGGTAT GGTTTCTCCA TTCAACCACC

111057493 GTCAACATTC CTCAATACAT CAACATCTCA GCTGACATTG AAGCAGTCTA CTCAATGCAG TCCAACAGCA AATTTGGTAT GGTTACTCCA TTCAACCACC

111061289 GTCAACATTC CTCAATACAT CAACATCTCA GCTGACATTG AAGCAGTCTA CTCAATGCAG GCCAACAGCA AATTTGGTGT GGTTGCTCCA TTCAACCACC

3701 3800

111061268 ATGAGTACTA TGCCAGTGTT GAGAAGAACA TGCAGTGCTA TTTGGCTGTT CAGACAGAGG CAAACATTGA CTTGGAAAAC AATGAGGTCG AATTCACAGT

111061279 ATGAGTACTA TGCCAGTGTT GAGAAGAACA TGCAATTCTA TATGGCTGTT CAGACAGAGG CAAACATTGA CTTGGAAAAC AGCGAGGTCG AATTCACAGT

111057493 ATGAGTACTA TGCCAGTGTT GAGAAGAACA TGCAATTCTA TATGGCTGTT CAGACAGAGG CAAACATTGA CTTGGAAAAC AACGAGGTCG AATTCACAGT

111061289 ATGAGTACTA TGCCAGTGTT GAGAAGAACA TGCAATTCTA TATGGCTGTT CAGACAGAGG CAAACATTGA CTTGGAAAAC AACGAGGTCG AATTCACAGT

3801 3900

111061268 GCAGCCACTC AACAAGGAAG ACAAGCAGAA TGTGTTCCAG TACAGCTCAG TGCTCTACAC CACCAAGTCA AACATCCTCA ACTTCAACCC AGCTCTGCAG

111061279 GCAGCCACTC AACAAGGAAG ACAAGCAGAA TGTGTTCCAG TACAGCACAG TGCTCTACAC CACCAAGTCA AACATCCTCA ACTTCAACCC AGCTCTGCAG

111057493 GCAGCCACTC AACAAGGAAG ACAAACAGAA TGTGTTCCAG TACAGCTCAG TGCTCTACAC CACCAAGTCA AACATCCTCA ACTTCAACCC AGCTCTGCAG

111061289 GCAGCCACTC AACAAGGAAG ACAAGCAGAA TGTGTTCCAG TACAGCACAG TGCTCTACAC CACCAAGTCA AACATCCTCA ACTTCAACCC AGCTCTGCAG

3901 4000

111061268 GAGGATGGCA CTGAGAGAGT GCATGTTGGC AAAGCTAAAC AGATCCAGAT GAACTTTGGC AAGGAGAGCA CTGGATTCGC ATTTGAGGCC AACTACTGGA

111061279 GAGGATGGCA CTGAGAGAGT GCATGTTGGC AAAGCTAAAC AGATCCAGAT GAACTTTGGC AAGGAGAGCA CTGGATTCAC ATTTGAGGCC AACTACTGGA

111057493 GAGGATGGCA CTGAGAGAGT GCATGTTGGC AAAGCTAAAC AGATCCAGAT GAACTTTGGC AAGGAGAGCA CTGGATTCGC ATTTGAGGCC AACTACTGGA

111061289 GAGGATGGCA CTGAGAGAGT GCATGTTGGC AAAGCTGAAC AGATTGAGAT GAAATTTGGC AAGGAAAGCA CTGGATTCGC ATTTGAGGCC AGCTACTGGA

4001 4100

111061268 GTGAGAATGG CTTTGGAGAC TTTGCCAGCC TCTACAATGA AGTTAGCAAG TTTGATTTCC AGTCTGCAAT GACATCTCCC TGGGCTCAAG GCTCCATCAA

111061279 GTGAGAATGG CTTTGGAGAC TTTGCCAGCC TCTACAATGA AGTTAGCAAG TTTGATTTCC AGTCTGCAAT GACATCTCCC TGGGCTCAAG GCTCCATCAA

111057493 GTGAGAATGG CTTTGGAGAC TTTGCCAGCC TCTACAATGA AGTTAGCAAG TTTGATTTCC AGTCTGCAAT GACATCTCCC TGGGCTCAAA GCTCCATCAA

111061289 GTGAGAATGG CTATGGAGAC TTTGCCACAC TCTACAATGA AGTTAGCAAG TTTGATTTCC AGTCTGCAAT GACATCTCCC TGGGCTCAAG GCTCCCTCAA

4101 4200

111061268 CTCCAACAAC ATTACTGTTG CCTTCAATCC AAGGCAGAGC ACCTCTCAGG TTGCCAAATT TACTTTCTCC TATGCTGACA ACTCTGATGA CAACAATAAC

111061279 CTCCAACAAC ATTACTGTTG CCTTCAATCC AAGGCAGAGC ACCTCTCAGG TTGCCAAATT TACTTTCTCC TATGCTGACA ACTCTGATGA CAACAATAAC

111057493 CTCTAACAAC ATAACTGTTG CCTTCAATCC AAGGCAGAGC ACCTCTCAGG TTGCCAAGTT TACTTTCTCC TATGCTGACA ACTCTGATGA CAACAATAAC

111061289 CTCCAACAAC ATTACTGTTG CCTTCAATCC AAGGCAGAGC ACCTCTCAGG TTGCCAAGTT TACTTTCTCC TATGCTGAAA ACTCTGATGA CAACAATAAC

4201 4300

111061268 TCCCACAGTG GACATGACAG CAACAGCAGC AACAACAACA ATAACAACAG AGCAGACTAC TCTGATGCTC AGCCATCTTC CACTGCTGCC AACAGTCGTT

111061279 TCCCACAGTG GACATGACAG CAACAGCAGC AACAACAACA ATAACAACAG AGCAGACTAC TCTGATGCTC AGCCATCTTC CACTGCTGCC AACAGTCGTT

111057493 TCCCACAGTG GACATGACAG CAACAACAGC AACAACAACA ATAACAACAG AGCAGACTAC ACTGATGCTC AGCCATCTTC CACTGCTGCC AACAGTCGTT

111061289 TCCCACAGTG GACATGACAG CAACAGCAGC AACAACAACA ATAACAACAG GGCAGACTAC TCTGATGCTC AGCCATCTTC CACAGCTGCC AACAGTCGTT

4301 4400

111061268 CCAGACAGAA TGAGTTCCTG CGCAAGGCTG CTGCTGGCAT CTCAGGTGCT GATGCTATGG TTGTTGATGT GTCTGCTAGA TTCCAAGACA GCCATGGTCA

111061279 CCAGACAGAA TGAATTCCTG CGCAAGGCTG CTGCTGGCAT CTCAGGTGCT GATGCTATGG TTGTTGATGT GTCTGCTAGA TTCCAAGACA GCCATGGTCA

111057493 CCAGACAGAA TGAATTCCTG CGCAAGGCTG CTGCTGGCAT CTCAGGTGCT GATGCTATGG TTGTTGATGT GTCTGCTAGA TTCCAAGACA GCCATGGTCA

111061289 CCAGACAGAA TGAATTCCTG CGCAAGGCTG CTGCTGGCAT CTCAGGTGCT GATGCTGTGG TTGTTGATGT GTCTGCTAGA TTCCAAGGCA GCCATGGTCA

4401 4500

111061268 ATCCAATGCC CAGTATGTAG CCACTGTAGC AATGGCCAAC AGTGATGCTT CTCCAAACGC TCGTATGCTG TTCTTTGCAT CAATGAACCC AGCTAACTCT

111061279 ATCCAATGCC CAGTATGTAG CCACTGTAGC AATGGCCAAC AGTGATGCTT CTCCAAACGC TCGTATGCTG TTCTTTGCAT CAATGAACCC AGCTAACTCT

111057493 ATCCAATGCC CAGTATGTAG CCACTGTAGC AATGGCCAAC AGTGATGCTT CTCCAAACGC TCGTATGCTG TTCTTTGCAT CAATGAACCC AGCTAACTCT

111061289 ATCCAATGCC CAGTATGTAG CCACTGTAGC AATGGCCAAC AGTGATGCTT CTTCAAACGC TCGTATGCTG TTCTTTGCAT CAATGAACCC AGCTAACTCT

4501 4600

111061268 GACTCCAAGG CCCAGGTGTG TGCTGCAGCT GCCAGTAACT TCCCCAATGT GCCACTCATG AACTTCCATG ATGCCCTCAA GGCAAACCCT ACTTCCCGCA

111061279 GACTCCAAGG CCCAGGTGTG TGCTGCAGCT GCCAGTAACT TCCCCAATGT GCCACTCATG AACTTCCACG ATGCCCTCAA GGCAAACCCT ACTTCCCGCA

111057493 GACTCCAAGG CCCAGGTGTG TGCTGCAGCT GCCAGTAACT TCCCCAATGT GCCACTCATG AACTTCCATG ATGCCCTCAA GGCAAACCCA ACTTCCCGCA

111061289 GACTCCAAGG CCCAGGTGTG TGCTGCAGTT GCCAGTAACT TCCCCAATGT GCCACTCATG AACTTCCTTG ATGCCCTTAA GGCAAACCCA ACTTCCCACA

4601 4700

111061268 TCAGTGCTGA CATTGCCTTT GGAGCACAAT GCAATGCTGG AGGACACATC CATGCTGATG CCAAGTTGTC ACAGACCCAG GAATTCCAGG AGTATACAAA

111061279 TCAGTGCTGA CATTGCCTTT GGAGCACAAT GCAATGCTGG AGGACACATC CATGCTGATG CCAAGTTGTC ACAGACCCAG GAATTCCAGG AGTATGCAAA

111057493 TCAGTGCTGA CATTGCCTTT GGTGCACAAT GCAATGCTGG AGGACACATC CATGCTGATG CCAAGTTGTC ACAGACCCAG GAATTCCAGG AGTATGCAAA

111061289 TCAGTGCTGA CATTGCCTTT GGAGCACAAT GCAATGCTGG AGGACACATC CATGCTGATG CCAGGTTGTC ACAGACCCAG GAATTCCAGG AGTATGCAAA

4701 4800

111061268 GAGCCGTCCA ATGGCCAAGA AATGCTTCCA GTTGATGGAG AAGGGCCAGG CTCTTGAATA TGCTTGTCAG AATGCCACCA AGGTCGCCAA CATGCTCAAC

111061279 GAGCCGTCCA ATGGCCAAGA AATGCTTCCA GTTGATGGAG AAGGGCCAGG CTCTAGAATA TGCTTGCCAG AATGCCACCA AGGTCGCCAA CATGCTCAAC

111057493 GAGCCGTCCA ATGGCCAAGA AATGCTTCCA ATTGATGGAG AAGGGCCAGG CTCTTGAATA TGCTTGCCAG AATGCCACCA AGGTCGCCAA CATGCTCAAC

111061289 GAGCCGTCCA ATGGCCAAGA AATGCTTCCA GTTGATGGAG AAGGGCCAGG CTCTTGAATA TGCTTGCCAG AATGCCACCA AGGTCGCCAA CATGCTCAAC

4801 4900

111061268 AACTACGAAG TCTCTGTCAA GTATGATAGA GTATCAAGTG TCTTCAAGAA TGTCACCTAC AGCATCTACT CAGCTCTGGC TCAAGCTGCC TACCCATACC

111061279 AACTACGAAG TCTCTGTCAA ATATGATAGA GTATCAAGTG TCTTCAAGAA CGTCACCTAC AGCATCTACT CAGCTCTGGC TCAAGCTGCC TACCCATACC

111057493 AACTACGAAG TCTCTGTCAA GTATGATAGA GTATCAAGTG TCTTCAAGAA CGTCACCTAC AGCATCTACT CAGCTCTGGC TCAAGCTGCC TACCCATACC

111061289 AACTACGATG TCTCAGTCAA GTATGATAGA GTACCAAATG CCCTCAAGAA CATCACCTAC AGCATCTACT CAGCTCTGGC TCAAGCTGCC TACCCATACC

4901 5000

111061268 ACAGTGAAAA CATGTTCAGC CAGAACAGCA ATCCTTCTGG CAAGATTGAT CTCAATGCCA GGTTCAACTA CAACCTCCGT TACTTCAATG CTTCAATCAA

111061279 ACAGTGAAAA CATGTTCAGC CAGAACAGCA ATCCTTCTGG CAAGATTGAT CTCAATGCCA GGTTCAACTA CAACCTCCGT TACTTCAATG CTTCAATCAA

111057493 ACAGTGAAAA CATGTTCAGT CAGAACAGCA ATCCTTCTGG CAAGATTGAT CTCAATGCCA GGTTCAACTA CAACCTCCGT TACTTCAATG CTTCAATCAA

111061289 ACAATAAGAA CATGTTCAGT CAGAACAGCA ATCCTGCTGG CAGGATTGAA GCCAATGTCA GGTTCAACAA CAACCTCCAT TACTTCAATG CTTCAATCAA

5001 5100

111061268 CACTCCATTC TTCTCTGCCA ATGTCAAGAA TGTTGAAGTA CACCATGCTC TCAGACCTCT GGTGATCTTC CATCCATCAC TCAACTCACT TGAGCTCATG

111061279 CACTCCATTC TTCTCTGCCA ATGTCAAGAA TGTTGAAGTA CACCACACTC TCAGACCTCT GGTGATCTTC CATCCATCAC TCAACTCACT TGAGCTCATG

111057493 CACTCCATTC TTCTCTGCCA ATGTCAAGAA TGTTGAAGTA CACCATGCTC TCAGACCTCT GGTGATCTTC CATCCATCAC TCAACTCACT TGAGCTCATG

111061289 CACTCCATTC TTCTCTGGCA ATGTCAAGAA TGTTGAAGTG GACCCTGCTC TCAGACCTCT GGTGATCTTC CATCCATCCC TCAACTCATT TGAGCTCATG

5101 5200

111061268 TCCTACAATG AGAACTATGA TTACCCAACA TGTTCTGTCA GCAAGAACTC CATCAGCACA TTCGACAACA AGACCTACTC AGCTGACCTT GAAGGCTGGC

111061279 TCCTACAATG AGAACTACGA TTACCCAACA TGTTCTGTCA GCAAGAACTC CATCAGCACA TTCGACAACA AGACCTACTC AGCTGACCTT GAAGGCTGGC

111057493 TCCTACAATG AGAACTATGA TTACCCAACA TGTTCTGTCA GCAAGAACTC CATCAGCACA TTCGACAACA AGACCTACTC AGCTGACCTT GAAGGCTGGC

111061289 TCCTACAATG AGAACTACGA TTACCCAACA TGTTCTGTCA GCAAGAACTC CATCAGCACA TTCGACAACA AGACCTACTC AGCTGACCTT GAAGGCTGGC

5201 5300

111061268 ATGTGATGTT TGCCTCCACT CCAAAGAACT ACAACGACAA CTCTGGAAGA TACAGTGCCA GCAACAGCCA GTCCAACAGC TTCTACAAGT ACAAGAAGGT

111061279 ATGTGATGTT TGCCTCCACT CCAAAGAACT ACAACGACAA CTCTGGAAGA TACAGTGCCA GCAACAGCCA GTCCAACAGC TTCTACAAGT ACAAGAAGGT

111057493 ATGTGATGTT TGCCTCCACT CCAAAGAACT ACAACGACAA CTCTGGAAGA TACAGTGCCA GCAACAGCCA GTCCAACAGC TTCTACAAGT ACAAGAAGGT

111061289 ATGTGATGTT TGCCTCCACT CCAAAGAACT ACAACGACAA CTCTGGAAGA TACAGTGCCA GCAACAGCCA GTCCAACAGC TTCTACAAGT ACAAGAAGGT

5301 5400

111061268 TGTTGTCTTG GCCAAGAATG CTGGATCTCA GCGCAAGGCT GTCAAGATGC TGTTGGGAGA GAATGTCATT GACATCAACC CCTCTGGCTC CGAGTCAAGT

111061279 TGTTGTCTTG GCCAAGAACG CCGGATCTCA GCGCAAGGCT GTCAAGATGC TGTTGGGAGA GAATGTCATT GACATCAACC CCTCTGGCTC CGAGTCAAGT

111057493 TGTTGTCTTG GCCAAGAACG CCGGATCTCA GCGCAAGGCT GTCAAGATGC TGTTGGGAGA GAATGTCATT GACATCAACC CCTCTGGCTC CGAGTCAAGT

111061289 TGTTGTCTTG GCCAAGAACG CCGGATCTGA GCGCAAGGCT GTCAAGATGC TGTTGGGAGA GAATGTCATT GACATCAACC CCTCTGGCTC CGAGTCAAGT

5401 5500

111061268 GACAACAGCC CCAATGCCAA TGTCCAGGTC AATGGAAACA AGGTGCAGAT TGCCAACAAC AGAATGGCCA GTTTCGATGA CTTTGATGGT GAGACCCTCG

111061279 GACAACAGCC CCAACGCCAA TGTCCAGGTC AATGGAAACA AGGTGCAGAT TGCCAACAAC AGGATGGCCA GTTTCGATGA CTTTGATGGT GAGACCCTCG

111057493 GACAACAGCC CCAACGCCAA TGTCCAGGTC AATGGAAACA AGGTGCAGAT TGCCAACAAC AGGATGGCCA GTTTCGATGA CTTTGATGGT GAGACCCTCG

111061289 GACAACAGCC CCAACGCCAA TGTCCAGGTC AATGGAAACA AGGTGCAGAT TACCAACAAC AGGATGACCA GTTTCGATGA CTTTGATGGT GAGACCCTCG

5501 5600

111061268 TTGAGATCTC TGTCACAGAC AATGGAGAGG TGCAGGTTCA GTCTTCATCC CATGGCATTG CTGTCTACCA CGATGGTGCC AACTTCATCA TTGATGCTGA

111061279 TTGAGATCTC TGTCACAGAC AATGGAGAGG TGCAGGTTCA GTCTTCATCC CATGGCATTG CTGTCTACCA CGATGGTGCC AACTTCATCA TTGATGCTGA

111057493 TTGAGATCTC TGTCACAGAC AATGGAGAGG TGCAGGTTCA GTCTTCATCC CATGGCATTG CTGTCTACCA CGATGGTGCC AACTTCATCA TTGATGCTGA

111061289 TTGAGATCTC TGTCACAGAC AATGGAGAGG TGCAGGTTCA GTCTTCATCC CATGGCATTG CTGTCTACCA CGATGGTGCC AACTTCATCA TTGATGCTGA

5601 5700

111061268 CAGCTACCAC AGAGGTGAGG TGCGTGGTCT CTGCGGTACC TACTCTGGTG ACAAGTACAC TGACTTCACC ACACCCAACA AGTGCATTAT GAGGGAGGCT

111061279 CAGCTACCAC AGAGGTGAGG TGCGTGGTCT CTGTGGTACC TACTCTGGTG ACAAGTACAC TGACTTCACC ACACCCAACA AGTGCATTAT GAGGGAGGCT

111057493 CAGCTACCAC AGAGGTGAGG TGCGTGGTCT CTGCGGTACC TACTCTGGTG ACAAGTACAC TGACTTCACC ACACCCAACA AGTGCATTAT GAGGGAGGCT

111061289 TAGCTACCAC AGAGGTGAGG TACGTGGTCT CTGCGGTACC TACTCTGGTG ACAAGTACAC TGACTTCACC ACACCCAATA AGTGCATTAT TAGGGAGGCT

5701 5800

111061268 AGACTGTTTG CAGCCACCTA TGCTCTGCCA GGCAGCAGCA ACAGCAACGT TGAACAGCTC AAGAGGCAGG CTGACCAGAT GACTTGCTTC AGGCGCCGTC

111061279 AGACTGTTTG CAGCCACCTA TGCTCTGCCA GGCAGCAGCA ACAGCAACGT TGAACAGCTC AAGAGGCAGG CTGACCAGAT GCCTTGCTTC AGGCGCCGTC

111057493 AGACTGTTTG CAGCCACCTA TGCTCTGCCA GGCAGCAGCA ACAGCAACGT TGAACAGCTC AAGAGGCAGG CTGACCAGAT GCCTTGCTTC AGGCGCCGGC

111061289 AGACTGTTTG CAGCCACCTA TGCTCTGCCA GGTAGCAGCA ACAGCAACGT TGAACAACTC AAGAGGCAGG CTGACCAGAT GCCTTGCTTG AGGCGCCGTC

```
          5801                                                                                                  5900
111061268 ACATCTTTGC TAATGTTATT ACTTCCAACG ACTATGACAG AAGCAGCAGC AGCAGCAGCA GCAGCAACAG GAACAACAAC AGGAACAACA ACAAGAACAA
111061279 ACATCTTTGC TAATGTCATC ACTTCTAATG ACTATGACAG AAGCAGCAGC AACAGCA--- ------ACA- --ACAAC--- ------CACA GC--------
111057493 ACATCTTTGC TAATGTCATC ACTTCCAATG ACTATGACCG AAGCAGCAGC AGCAGCA--- ------ACAG GAACAACAAC AAGAACCACA GC--------
111061289 ACATCTTTGC TGATGTCATC ACTTCCAACG ACTATGACAG AAGCAGCAGC AGCAGCA--- ------GCAG CAGCAGCAAC AGGAACAACA ACAAGAACAA
          5901                                                                                                  6000
111061268 CAGGAGCAAC AACAACAGTT CAGAGAGACT CGCAAACCCA ACCAAGCTTA TCCAGGACGT CAAGAACAAT GGTGACCAAG TCTGCATCAG TATTCGCCCA
111061279 ---------- ---------T CAGAGAGGTT CATCAACCCA ACGAAACTGG TGCAGGACAT CAAGAACAAC CTTGACCGTC TTTGCTTCAG TATTCACCCA
111057493 ---------- ---------T CAGAGAGACT TGCCAACCCA ACCAAACTTG TGCAGGACAT CAAGAACAAT GGTGACCAGG TCTGCTTCAG TATTCACCCA
111061289 CAGGAACAAC AATAACAGTT CAGAATTGCG CACCAACCCA ACCAAACTGG TGCAGGACAT CAAGAACAAT GGTGACCATG TCTGCTTCAG TATTCACCCA
          6001                                                                                                  6100
111061268 GTCCCCAAGT GCCAGAAAGG CTTCTCCCCA GCTGGATCAT CTGAGAAAGA GGTTGACTAC GTCTGTATGA GTCACGGCAA GAATGCCCAA TTTTGGATCA
111061279 GTCCCCAAAT GCCAGAGCGG CTTCTCCCCA GCTGAATCAT CTGAGAAAGA GTTTCAGTAC TTCTGCATGG ACCAGGGCAA GAACGCCCAA TATTGGGCCA
111057493 GTCCCCAAGT GCCAGAGTGG CTTCTCCCCA GCTGGATCAT CTGAAAAGGA GGTTCAGTAC TTCTGCATGG GCCAGGGCAA GAACTCCGAA TATTGGGTTG
111061289 GTCCCCAAGT GCCAGAGTGG CTTCTCCCCA GCTGAATCAT CTGAGAAGGA GGTTCAGTAC CTGTGCATCA GCAAGGGCAA GAACGCCGAA TATTGGGTTG
          6101                                                                                               6198
111061268 ATCAGATCTT CCAAGGTGGA TATGTCAAGC TTGAACAGAA GCAGCATAAT GCCACATTCA TGAAGAACAT TCCACAGAGA TGTGTCAGAG ACAACTAA
111061279 ATCAAATCCG CAACGGTGGA TTCGTCAACC TTGAACAGAA GCAGCCTAAT GCTACATTCA AGAAGAACAT CCCAAAGAGT TGTGTCAGAG ACAACTAG
111057493 GTCAGATCAT CAACGGTGGA TTCGTCAACC TTGGACAGAA GCAGCCTAAT GCCACATTCA AGAAGAGCGT CCCAAAGAGT TGTGTTAGAG ACAACTAA
111061289 GTCAGATCAG CAATGGTGGA TTCGTCAACC TTGGACAGAA GCAGCCTAAT GCCACATTCA AGAAGAGCGT CCCAAAGAGT TGTGTTAGAG ACAACTAA
```
